# Phase 1b study to assess the safety, tolerability, and clinical activity of pamiparib in combination with temozolomide in patients with locally advanced or metastatic solid tumors

**DOI:** 10.1002/cam4.7385

**Published:** 2024-07-05

**Authors:** Agostina Stradella, Melissa Johnson, Sanjay Goel, Haeseong Park, Nehal Lakhani, Hendrik‐Tobias Arkenau, Matthew D. Galsky, Emiliano Calvo, Vicente Baz, Victor Moreno, Omar Saavedra, Stephen J. Luen, Song Mu, Qiting Wan, Victoria Chang, Wa Zhang, Minal Barve

**Affiliations:** ^1^ Institut Català d'Oncologia–Hospital Duran I Reynals, L'Hospitalet de Llobregat Catalunya Spain; ^2^ Sarah Cannon Research Institute, Tennessee Oncology, PLLC Nashville Tennessee USA; ^3^ Rutgers Robert Wood Johnson Medical School New Brunswick New Jersey USA; ^4^ Washington University School of Medicine St. Louis Missouri USA; ^5^ Dana‐Farber Cancer Institute Boston Massachusetts USA; ^6^ START Midwest Grand Rapids Michigan USA; ^7^ Sarah Cannon Research Institute, UCL Cancer Institute, University College London London UK; ^8^ The Tisch Cancer Institute Icahn School of Medicine at Mount Sinai New York New York USA; ^9^ START Madrid‐HM CIOCC, Centro Integral Oncológico Clara Campal, Hospital Universitario HM Sanchinarro, Calle Oña Madrid Spain; ^10^ Hospital Universitario Virgen Macarena Seville Spain; ^11^ START Madrid‐FJD Fundacion Jimenez Diaz University Hospital Madrid Spain; ^12^ Vall d'Hebron Institute of Oncology Barcelona Spain; ^13^ Division of Cancer Research Peter MacCallum Cancer Centre Melbourne Victoria Australia; ^14^ The Sir Peter MacCallum Department of Medical Oncology The University of Melbourne Melbourne Victoria Australia; ^15^ BeiGene, USA Inc Ridgefield Park New Jersey USA; ^16^ BeiGene (Beijing) Co., Ltd Beijing China; ^17^ BeiGene, USA Inc San Mateo California USA; ^18^ Mary Crowley Cancer Research Dallas Texas USA

**Keywords:** DNA repair, clinical trials, target therapy, biomarkers

## Abstract

**Background:**

Pamiparib is a potent, selective, poly (ADP‐ribose) polymerase 1/2 inhibitor that demonstrates synthetic lethality in cells with breast cancer susceptibility gene mutations or other homologous recombination deficiency. This two‐stage phase 1b study (NCT03150810) assessed pamiparib in combination with temozolomide (TMZ) in adult patients with histologically confirmed locally advanced and metastatic solid tumors.

**Methods:**

Oral pamiparib 60 mg was administered twice daily. During the dose‐escalation stage, increasing doses of TMZ (40–120 mg once daily pulsed or 20–40 mg once daily continuous) were administered to determine the recommended dose to be administered in the dose‐expansion stage. The primary objectives were to determine safety and tolerability, maximum tolerated/administered dose, recommended phase 2 dose and schedule, and antitumor activity of pamiparib in combination with TMZ. Pharmacokinetics of pamiparib and TMZ and biomarkers were also assessed.

**Results:**

Across stages, 139 patients were treated (dose escalation, *n* = 66; dose expansion, *n* = 73). The maximum tolerated dose of TMZ, which was administered during dose expansion, was 7‐day pulsed 60 mg once daily. The most common treatment‐emergent adverse events (TEAEs) were anemia (dose escalation, 56.1%; dose expansion, 63.0%), nausea (dose escalation, 54.5%; dose expansion, 49.3%), and fatigue (dose escalation, 48.5%; dose expansion, 47.9%). In the dose‐escalation stage, four patients experienced dose‐limiting toxicities (three neutropenia and one neutrophil count decreased). No TEAEs considered to be related to study drug treatment resulted in death. Antitumor activity was modest, indicated by confirmed overall response rate (dose escalation, 13.8%; dose expansion, 11.6%), median progression‐free survival (3.7 and 2.8 months), and median overall survival (10.5 and 9.2 months). Administration of combination therapy did not notably impact pamiparib or TMZ pharmacokinetics.

**Conclusions:**

Pamiparib in combination with TMZ had a manageable safety profile. Further investigation of the efficacy of this combination in tumor types with specific DNA damage repair deficiencies is warranted.

## 1 INTRODUCTION

Poly (ADP‐ribose) polymerase (PARP) inhibitors are a class of anticancer agents that prevent the PARP family of proteins from repairing single‐strand DNA breaks, which accumulate and convert into double‐strand breaks during DNA replication.^1^ PARP inhibition can be fatal in cells that cannot undergo double‐strand DNA break repair and thus experience synthetic lethality (the two conditions combined are lethal but are not lethal independently).^2^ PARP inhibitors have demonstrated synthetic lethality in cells with breast cancer susceptibility gene 1/2 (*BRCA*1/2) mutations and in cells with homologous recombination deficiency (HRD).^2^, ^3^, ^4^ Some PARP inhibitors, including pamiparib, have also been shown to trap PARP1/2 proteins at DNA damage sites, which appears to potentiate their cytotoxic effect.^1^, ^5^ PARP inhibitors, including olaparib, rucaparib, niraparib, and talazoparib, are approved by the US Food and Drug Administration to treat a wide range of malignancies with deleterious *BRCA* mutations or HRD+ status, including ovarian, breast, prostate, and pancreatic cancers.^6^, ^7^, ^8^, ^9^

In addition to monotherapy use, PARP inhibitors have been investigated in combination with therapies that target DNA damage pathways.^2^ Temozolomide (TMZ) is a US Food and Drug Administration–approved alkylating agent that triggers DNA breaks by methylating guanine at the O^6^ position, leading to cell cycle arrest and apoptosis.^10^, ^11^ The base excision repair (BER) pathway is activated to repair TMZ‐induced DNA damage; however, PARP inhibition can interfere in BER‐mediated repair and potentiate antitumor effects.^10^ Preclinical models indicate that repeated treatment with TMZ and PARP inhibitors downregulates transcription and delays recovery of BER components in tumor cells, which may further sensitize cells to combination treatment.^10^, ^12^, ^13^ Clinical trials have investigated TMZ in combination with the PARP inhibitors olaparib,^14^ rucaparib,^15^ talazoparib,^16^ and veliparib^17^, ^18^, ^19^, ^20^, ^21^ for a variety of malignancies. Antitumor activity has been shown for the combination of TMZ plus rucaparib in metastatic melanoma (objective response rate [ORR] of 17.4%)^15^ and TMZ plus veliparib in relapsed/refractory acute myeloid leukemia or acute myeloid leukemia arising from aggressive myeloid malignancies (complete response [CR] rate of 16.7%).^20^ However, as with PARP inhibitor monotherapy,^6^, ^7^, ^8^, ^9^ hematological toxicities, including neutropenia and thrombocytopenia, were commonly reported.^14^, ^16^, ^17^, ^18^, ^19^, ^21^

Pamiparib (BGB‐290) is a potent and selective inhibitor of PARP1 and PARP2,^5^ and as monotherapy is approved in China for the treatment of germline *BRCA* mutation‐associated recurrent advanced ovarian, fallopian tube, or primary peritoneal cancer which has been previously treated with at least two lines of chemotherapy.^5^, ^22^, ^23^ Initial preclinical evidence established PARP‐DNA complex trapping with pamiparib and potent antitumor activity in multiple cancer cell types with *BRCA1/2* mutations or HRD.^5^, ^22^ Studies in mice and rats have shown strong brain penetration with pamiparib; furthermore, studies in mice have shown that pamiparib has higher drug exposure in the brain compared with other PARP inhibitors.^5^ Thus, pamiparib may be particularly attractive among PARP inhibitors for use in combination with agents such as TMZ when treating cancers that metastasize to the brain.

In phase 1 and 2 clinical studies (NCT02361723, NCT03333915, and NCT03575065), pamiparib monotherapy has demonstrated antitumor activity and induced durable responses in patients with ovarian and breast cancer.^24^, ^25^, ^26^, ^27^ Across these studies, pamiparib monotherapy resulted in an ORR of 27.3% in a phase 1a/b dose‐escalation/dose‐expansion study of patients with advanced or metastatic solid tumors^24^; 64.6% among patients with *BRCA1/2*‐mutated platinum‐sensitive ovarian cancer and 31.6% among patients with *BRCA1/2*‐mutated platinum‐resistant ovarian cancer in a phase 2 study^25^; and 38.2% among triple‐negative breast cancer (TNBC) patients with germline *BRCA1/2* mutations in a phase 2 study.^26^ Combination therapy of pamiparib with the programmed cell death protein‐1 checkpoint inhibitor tislelizumab also demonstrated antitumor response (ORR of 20.4%) in advanced solid tumors in a phase 1a/b clinical trial (NCT02660034).^7^ Pamiparib treatment has been generally well tolerated in patients with advanced solid tumors, with a safety profile similar to other PARP inhibitors in these populations.^24^, ^25^, ^26^, ^27^, ^28^

The current phase 1b study assessed the safety, tolerability, and clinical activity of pamiparib in combination with TMZ in patients with locally advanced and metastatic solid tumors. In order to limit hematological toxicities, a recent phase 1 dose‐escalation study of patients with advanced malignancies and wild‐type *BRCA* evaluated low doses of chemotherapy (TMZ or irinotecan) combined with a full dose of talazoparib.^16^ This study showed that the combination was reasonably well tolerated and had clinical activity.^16^ In our study, the initial dose‐escalation stage investigated a variety of TMZ dosing regimens to determine the maximum tolerated dose (MTD) and a recommended phase 2 dose (RP2D) of TMZ in combination with a fixed dose of pamiparib. The dose‐expansion stage of the study investigated this dose in patients with a variety of tumor types. Here, results are reported from both the dose‐escalation and dose‐expansion stages of the trial.

## 2 METHODS

### 2.1 Study Design and Treatment

This phase 1b open‐label, multicenter study (NCT03150810) evaluated combination therapy with pamiparib and TMZ in patients with locally advanced and metastatic solid tumors. The trial was conducted in accordance with the International Council for Harmonization Good Clinical Practice, the principles of the Declaration of Helsinki, and local laws and regulations. The protocol was approved by the relevant institutional review board/independent ethics committee of each center (see Data S1); prior to study activity, all patients provided written informed consent.

The study included two stages, a dose‐escalation stage and a dose‐expansion stage. For the entirety of the dosing period, pamiparib was administered orally, twice daily (BID), approximately every 12 h. TMZ was administered orally, once daily (QD), preferably in the morning. Co‐administration of pamiparib and TMZ was permitted.

The dose‐escalation stage had a modified 3 + 3 design with a fixed dose of pamiparib in combination with escalating doses of TMZ in either pulsed (arm A) or continuous (arm B) administration. The selection of the fixed dose of pamiparib was based on the results of a phase 1a/b dose‐escalation/dose‐expansion study in patients with advanced solid tumors, which established the RP2D of pamiparib as 60 mg (administered orally, BID).^24^ Based on the results of a phase 1 study evaluating talazoparib in combination with low‐dose TMZ,^16^ we used a range of TMZ doses starting from 40 to 120 mg/day (23 to 69 mg/m^2^/day) or higher, which corresponds to 15%–46% of its recommended dose (150 mg/m^2^/day) when administered daily for 5 days every 28 days. In addition, continuous dosing of TMZ was started at 40 mg/day and could potentially be increased up to 120 mg/day, which translates to 30% and 92%, respectively, of a daily dose of 75 mg/m^2^.

In arm A, continuous pamiparib (60 mg BID) was administered in combination with increasing doses of TMZ from 40 to 120 mg QD for Days 1 through 7 of each 28‐day cycle (7‐day pulsed). In arm B, pamiparib 60 mg BID was administered in combination with TMZ (20 or 40 mg) QD continuously during each 28‐day cycle. Escalation was carried out starting from the first dose to assess dose‐limiting toxicities (DLTs) up to the MTD. A minimum of three patients were enrolled in each cohort. If none of the first three evaluable patients enrolled in a given cohort experienced a DLT, dose escalation could proceed. If one of the first three evaluable patients enrolled in a given cohort experienced a DLT, additional patients (for a minimum of six evaluable patients) were enrolled in that cohort. If fewer than two of six evaluable patients in a given cohort experienced a DLT, escalation proceeded to the next‐higher dose level. If a DLT was observed in two or more of up to six patients, the MTD was exceeded, and dose escalation was stopped. If the MTD was exceeded at a given dose level, the next‐highest dose level at which fewer than one‐third of evaluable patients in a given cohort experienced a DLT (e.g., DLTs in fewer than two of six patients) was declared the MTD. Once the MTD was determined in arm A and all data available for arm B at that time were taken into consideration, further dose escalation was pursued for arm A by extending the time window of TMZ administration by 1 week. In this additional investigatory arm, continuous pamiparib 60 mg with 40 mg TMZ for Days 1–14 of the 28‐day cycle (14‐day pulsed) was administered.

During the dose‐expansion stage, six dose‐expansion cohorts were planned based on indication and/or HRD status (see details below in “Patients”). Pamiparib was administered continuously in combination with TMZ at the dose and schedule determined in the dose‐escalation phase. Each cohort was evaluated independently and could be closed due to lack of enrollment, antitumor activity, or other reasons.

### 2.2 Patients

Both stages of the study enrolled adults (≥18 years of age) with histologically or cytologically confirmed malignancies with advanced or metastatic disease who may have progressed on standard‐of‐care treatment. Patients had an Eastern Cooperative Oncology Group performance status (ECOG PS) ≤1 and adequate organ function. Patients must have had disease that was either evaluable (dose‐escalation cohort) or measurable (dose‐escalation and dose‐expansion cohorts) per Response Evaluation Criteria in Solid Tumors version (RECIST) version 1.1, except for patients with prostate cancer that had alternative measures for progression (Prostate Cancer Working Group 2 criteria, radiographic by modified RECIST v1.1, castration, or at least two new non‐measurable bone lesions). Patients agreed to provide archival tumor tissue or fresh biopsy.

The expansion stage enrolled patients with: cohort (1) ovarian cancer with either known or suspected deleterious mutations in *BRCA1* or *BRCA2* or documented positive HRD status (HRD+) who have received at least one line of platinum‐containing therapy in the advanced or metastatic setting and did not progress or have recurrent disease ≤6 months of the completion of the last platinum‐containing regimen; cohort (2) TNBC with either known or suspected deleterious mutations in *BRCA1* or *BRCA2* or documented HRD+, who had received up to one prior platinum‐containing treatment in any treatment setting and up to three prior lines of therapy in the advanced or metastatic setting; cohort (3) metastatic castration‐resistant prostate cancer with either known or suspected deleterious mutations in *BRCA1* or *BRCA2* or documented HRD+ who were either chemotherapy‐naïve or had previously received up to two taxane‐based chemotherapy regimens, with documented prostate cancer progression (no patients were enrolled in this cohort); cohort (4) extensive‐stage small cell lung cancer who had received up to two prior lines of therapy; cohort (5) gastric/gastroesophageal junction cancer who had received up to two prior lines of therapy; cohort (6) other HRD+ solid tumor types including non‐squamous non‐small cell lung cancer (NSCLC), squamous NSCLC, esophageal cancer, squamous head and neck cancer, or soft tissue sarcoma (undifferentiated pleomorphic sarcoma, leiomyosarcoma, malignant peripheral nerve sheath tumor, dedifferentiated liposarcoma, myxofibrosarcoma) who had received one to three prior lines of therapy. Patients with soft‐tissue sarcomas that were treatment naïve were also allowed if available standard‐of‐care first‐line therapy was not appropriate.

For cohorts 1–3, the patient must have undergone tissue screening at a central laboratory if HRD or *BRCA1/2* mutation status was unknown or had not been previously evaluated. For cohort 6, HRD status was prospectively analyzed at a central laboratory using the MyChoice CDx Plus HRD assay (Myriad Genetics). HRD+ was specifically defined as genomic instability score (GIS) ≥33 regardless of *BRCA1/2* mutation status, which was determined from the exploratory biomarker analysis in the dose‐escalation and dose‐expansion (cohorts 1–5) stages.

Key exclusion criteria were hypersensitivity to any TMZ component or dacarbazine; prior treatment with a PARP inhibitor; prior treatment with chemotherapy, biologic therapy, immunotherapy, or investigational agent ≤3 weeks prior to treatment initiation; refractory to platinum‐based therapy (for patients in the dose‐expansion phase only), Grade ≥2 unresolved acute effects from prior therapy; and diagnosis of other malignancy. Full eligibility criteria are provided in the Data S1.

### 2.3 Objectives

The primary objectives of this study were to determine the safety and tolerability of oral pamiparib in combination with TMZ (pulsed and continuous), to determine the MTD or maximum administered dose for pamiparib with TMZ, to select the RP2D and schedule, and to determine the antitumor activity of pamiparib in combination with TMZ. The secondary objective was to characterize the pharmacokinetics (PK) of pamiparib with TMZ. Exploratory objectives included evaluation of candidate biomarkers of response, resistance, or disease progression in tumor tissue and peripheral circulation.

### 2.4 Procedures and Assessments

The incidence, nature, and severity of adverse events (AEs) were graded according to the National Cancer Institute Common Terminology Criteria for Adverse Events (NCI CTCAE), v4.03. Safety was assessed at every site visit (weekly for cycle 1, biweekly for cycles 2–5, and then every 28 days for subsequent cycles). Each cycle was 28 days and included assessment of AEs, physical examination, vital signs, and clinical laboratory tests. AEs were coded using MedDRA v25.0 or higher. A treatment‐emergent adverse event (TEAE) was defined as an AE with an onset date or worsening in severity from baseline on or after the first dose of study drug until 30 days after the last dose of pamiparib or initiation of new anticancer therapy. The safety population included all patients who received at least one dose of pamiparib or TMZ. The DLT‐evaluable population included patients who received ≥70% of each study drug or had <70% of the scheduled dose who experienced a DLT event during the assessment window (first cycle of 28 days) in the dose‐escalation stage. A DLT was defined as one of the following toxicities occurring during the DLT assessment window and considered by the investigator to be related to pamiparib: Grade 4 anemia, Grade ≥4 neutropenia lasting >7 days, Grade ≥3 febrile neutropenia, Grade ≥3 thrombocytopenia with clinically significant bleeding, Grade ≥4 thrombocytopenia lasting >7 days, Grade ≥3 total bilirubin or hepatic transaminases or aspartate aminotransferase, or Grade ≥3 non‐hematologic, non‐hepatic major organ AE.

Clinical activity was evaluated by ORR assessed by the investigator using RECIST v1.1 (except for patients with prostate cancer who were evaluated according to Prostate Cancer Working Group 2 criteria and patients with ovarian cancer who may be assessed based on Gynecological Cancer Intergroup criterial). Other efficacy endpoints included duration of response (DoR), disease control rate (DCR, defined as patients with a best overall response of CR, partial response [PR], or stable disease [SD]), progression‐free survival (PFS), and overall survival (OS). Tumor imaging using computed tomography (preferred) or magnetic resonance imaging was performed at screening and every 8 weeks (±1 week) from cycle 1 Day 1 until disease progression. The efficacy‐evaluable population included patients from the safety population with evaluable disease in the dose‐escalation phase or measurable disease in the dose‐expansion phase at baseline and had at least one postbaseline tumor assessment, unless the patient discontinued treatment due to clinical progression or death prior to tumor assessment.

PK of pamiparib and TMZ were assessed in blood samples collected at various timepoints following administration (pamiparib: ≤30 min before dosing, 1 h ± 15 min, 2 h ± 30 min, and 4 h ± 30 min after pamiparib dose on cycle 1 Day 1 and cycle 1 Day 15; TMZ: on cycle 1 Day 1 and Day 7 at ≤30 min before dosing and 1 h ± 15 min after TMZ dose). Parameters for pamiparib included the maximum observed plasma concentration (C_max_), lowest concentration reached before the next administered dose (C_trough_), half‐life (T_1/2_), and time to reach peak plasma concentration (T_max_). Biomarkers were analyzed in blood samples, processed into serum, plasma, and cell fractions for the analysis of germline mutations and circulating markers. Available archival or fresh tumor tissue samples were analyzed at a central laboratory. Tumor tissue was sent either as a formalin‐fixed, paraffin‐embedded block with tumor tissue (preferred) or as approximately 10 unstained slides.

### 2.5 Statistical Analyses

Data were summarized by dose level for the dose‐escalation stage and by cohort for the dose‐expansion stage. Descriptive statistics were used to describe anticancer activities and tolerability, with confidence intervals to describe precision for point estimates. No formal hypothesis testing was planned for this study. Time‐to‐event variables (PFS, DoR, and OS) were estimated using the Kaplan–Meier method; medians were presented with two‐sided 90% confidence intervals using a generalized Brookmeyer and Crowley method. PFS and OS at 6 months were estimated using the Kaplan–Meier method, with 90% confidence intervals constructed using Greenwood's formula.

## 3 RESULTS

### 3.1 Patient Characteristics

**FIGURE 1:**
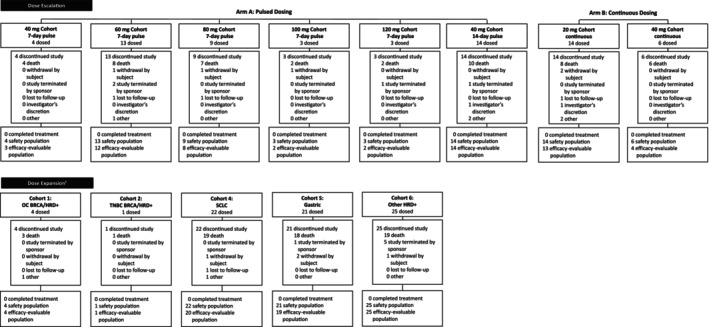
Patient disposition. There were 63 patients screened for the study who were not enrolled ^‡^No patients were enrolled in cohort 3 (metastatic castration‐resistant prostate cancer). BRCA, breast cancer susceptibility gene; HRD, homologous recombination deficiency; OC, ovarian cancer; SCLC, small cell lung cancer; TNBC, triple‐negative breast cancer.

Between July 12, 2017, and December 8, 2021, 139 patients were enrolled at 20 study centers in the US, UK, Spain, and Australia. The 139 patients treated were followed for a median of 9.1 months. In the dose‐escalation stage, 66 patients were treated and included in the safety population and 58 patients were included in the efficacy‐evaluable population (Figure 1). In the dose‐expansion stage, 73 patients were treated and included in the safety population and 69 patients were included in the efficacy‐evaluable population (Figure 1).

In both the dose‐escalation and ‐expansion stages, the safety patient population was mostly White (69.7% and 80.8%, respectively) and the median age was similar (66.5 and 61.0 years, respectively; Table 1). All patients had ECOG PS of 0 or 1 (ECOG PS of 1 in 75.8% of dose‐escalation patients and 68.5% of dose‐expansion patients). All patients had received at least one line of therapy and were heavily pretreated with a median of 3.5 (range, 1–10) prior regimens in the dose‐escalation stage and two (range, 1–5) in the dose‐expansion stage. Cohort 3 (metastatic castration‐resistant prostate cancer) was not initiated and, as such, no patients were enrolled in this group. In the dose‐escalation stage, 10 patients (15.2%) were known to be HRD+ and eight patients (12.1%) were known to have a tumor *BRCA* mutation. In the dose‐expansion stage, 30 patients (41.1%) were known to be HRD+ and six patients (8.2%) were known to have tumor *BRCA* mutation (Table 1). Across stages and treatment groups, the most common reason patients discontinued the study was death (107 patients, 77.0%) (Figure 1).

**TABLE 1:** Baseline demographics and clinical characteristics.

	Dose escalation									Dose expansion^a^					
	Arm A: Pulsed						Arm B: Continuous		Total						
	PAM 60 mg + TMZ 40 mg 7‐day pulse (*n* = 4)	PAM 60 mg + TMZ 60 mg 7‐day pulse (*n* = 13)	PAM 60 mg + TMZ 80 mg 7‐day pulse (*n* = 9)	PAM 60 mg + TMZ 100 mg 7‐day pulse (*n* = 3)	PAM 60 mg + TMZ 120 mg 7‐day pulse (*n* = 3)	PAM 60 mg + TMZ 40 mg 14‐day pulse (*n* = 14)	PAM 60 mg + TMZ 20 mg cont (*n* = 14)	PAM 60 mg + TMZ 40 mg cont (*n* = 6)	All Patients (*n* = 66)	Cohort 1: OC *BRCA*/HRD+ (*n* = 4)	Cohort 2: TNBC *BRCA*/HRD+ (*n* = 1)	Cohort 4: SCLC (*n* = 22)	Cohort 5: gastric (*n* = 21)	Cohort 6: other HRD+ (*n* = 25)	All patients (*n* = 73)
Median age (range), years	65.0 (55–69)	62.0 (38–70)	72.0 (65–86)	58.0 (51–72)	71.0 (50–76)	69.0 (48–82)	66.5 (48–82)	63.5 (61–83)	66.5 (38–86)	59.0 (57–62)	31.0	61.0 (46–77)	62.0 (26–75)	60.0 (35–73)	61.0 (26–77)
Age group, *n* (%)															
<65 years	2 (50.0)	8 (61.5)	0	2 (66.7)	1 (33.3)	5 (35.7)	5 (35.7)	3 (50.0)	26 (39.4)	4 (100)	1 (100)	13 (59.1)	13 (61.9)	21 (84.0)	52 (71.2)
≥65 years	2 (50.0)	5 (38.5)	9 (100)	1 (33.3)	2 (66.7)	9 (64.3)	9 (64.3)	3 (50.0)	40 (60.6)	0	0	9 (40.9)	8 (38.1)	4 (16.0)	21 (28.8)
Men, *n* (%)	1 (25.0)	6 (46.2)	4 (44.4)	1 (33.3)	2 (66.7)	8 (57.1)	7 (50.0)	4 (66.7)	33 (50.0)	0	0	14 (63.6)	13 (61.9)	15 (60.0)	42 (57.5)
Race, *n* (%)															
Asian	0	0	0	0	0	1 (7.1)	0	0	1 (1.5)	1 (25.0)	0	0	0	0	1 (1.4)
Black or African American	0	1 (7.7)	0	1 (33.3)	2 (66.7)	3 (21.4)	1 (7.1)	1 (16.7)	9 (13.6)	0	0	0	1 (4.8)	0	1 (1.4)
White	4 (100)	10 (76.9)	9 (100)	2 (66.7)	1 (33.3)	7 (50.0)	8 (57.1)	5 (83.3)	46 (69.7)	2 (50.0)	1 (100)	18 (81.8)	18 (85.7)	20 (80.0)	59 (80.8)
Other^b^	0	2 (15.4)	0	0	0	3 (21.4)	5 (35.7)	0	10 (15.2)	1 (25.0)	0	4 (18.2)	2 (9.5)	5 (20.0)	12 (16.4)
ECOG performance status, *n* (%)															
0	2 (50.0)	3 (23.1)	3 (33.3)	0	0	0	6 (42.9)	2 (33.3)	16 (24.2)	4 (100)	1 (100)	3 (13.6)	6 (28.6)	9 (36.0)	23 (31.5)
1	2 (50.0)	10 (76.9)	6 (66.7)	3 (100)	3 (100)	14 (100)	8 (57.1)	4 (66.7)	50 (75.8)	0	0	19 (86.4)	15 (71.4)	16 (64.0)	50 (68.5)
GIS^c^															
≥33, *n* (%)	1 (25.0)	1 (7.7)	2 (22.2)	0	0	2 (14.3)	3 (21.4)	0	9 (13.6)	4 (100)	0	0	1 (4.8)	25 (100)	30 (41.1)
<33, *n* (%)	2 (50.0)	6 (46.2)	1 (11.1)	1 (33.3)	2 (66.7)	6 (42.9)	2 (14.3)	3 (50.0)	23 (34.8)	0	0	6 (27.3)	8 (38.1)	0	14 (19.2)
Unknown, *n* (%)	1 (25.0)	6 (46.2)	6 (66.7)	2 (66.7)	1 (33.3)	6 (42.9)	9 (64.3)	3 (50.0)	34 (51.5)	0	1 (100)	16 (72.7)	12 (57.1)	0	29 (39.7)
Tumor BRCA status^c^, ^d^															
Mutation, *n* (%)	0	2 (15.4)	2 (22.2)	0	0	2 (14.3)	2 (14.3)	0	8 (12.1)	1 (25.0)	1 (100)	0	0	4 (16.0)	6 (8.2)
Wildtype, *n* (%)	2 (50.0)	4 (30.8)	2 (22.2)	1 (33.3)	1 (33.3)	5 (35.7)	3 (21.4)	3 (50.0)	21 (31.8)	3 (75.0)	0	6 (27.3)	10 (47.6)	21 (84.0)	40 (54.8)
Unknown, *n* (%)	2 (50.0)	7 (53.8)	5 (55.6)	2 (66.7)	2 (66.7)	7 (50.0)	9 (64.3)	3 (50.0)	37 (56.1)	0	0	16 (72.7)	11 (52.4)	0	27 (37.0)
Confirmed HRD positive,^e^ *n* (%)	0	1 (7.7)	3 (33.3)	0	1 (33.3)	1 (7.1)	3 (21.4)	1 (16.7)	10 (15.2)	4 (100)	1 (100)	0	0	25 (100)	30 (41.1)
Median time from initial diagnosis to study entry (range), years	3.8 (2.4–12.8)	4.1 (0.3–24.9)	2.8 (1.8–14.9)	2.7 (1.0–23.2)	6.0 (1.1–15.6)	4.4 (0.7–14.3)	4.5 (1.0–48.0)	2.1 (0.8–10.6)	3.5 (0.3–48.0)	5.6 (2.9–6.8)	0.9	0.8 (0.3–7.0)	1.3 (0.5–5.5)	1.4 (0.4–5.7)	1.3 (0.3–7.0)
Median no. of prior regimens (range)	5.5 (4–7)	3.0 (1–10)	4.0 (3–9)	4.0 (1–9)	5.0 (2–10)	3.0 (1–8)	3.0 (1–8)	2.5 (1–7)	3.5 (1–10)	3.0 (1–5)	1.0	2.0 (1–3)	2.0 (1–4)	2.0 (1–4)	2.0 (1–5)

Abbreviations: *BRCA*, breast cancer susceptibility gene; cont, continuous; ECOG, Eastern Cooperative Oncology Group; GIS, genomic instability score; HRD, homologous recombination deficiency; OC, ovarian cancer; PAM, pamiparib; SCLC, small cell lung cancer; TMZ, temozolomide; TNBC, triple‐negative breast cancer.

^a^ No patients were enrolled in cohort 3 (metastatic castration‐resistant prostate cancer).

^b^ Other race included other, unknown, not reported, and native Hawaiian or other Pacific islander.

^c^ GIS and *BRCA1/2* mutation status were analyzed based on central testing results.

^d^ Tumor *BRCA* status was determined through direct analysis of tumor samples or inferred from germline *BRCA* status. If a patient carried a germline *BRCA1/2* mutation, it was considered that their tumor also carried a *BRCA1/2* mutation.

^e^ The overall HRD status was confirmed based on the combined central testing results. In the case of ovarian cancer (i.e. ovarian cancer, fallopian cancer, and peritoneal cancer) and breast cancer, a tumor was considered HRD positive if it had either a GIS ≥42 or a *BRCA1/2* mutation. For patients from cohort 6, if their tumor had a GIS ≥33, it was determined as HRD positive. HRD status was not determined for the remaining indications.

In the dose‐escalation stage, the median pamiparib treatment duration was 2.58 months (range, 0.0–51.8 months) and the median pamiparib dose intensity per patient was 111.5 mg/day (range, 41.8–120.0 mg/day). In the dose‐expansion stage, pamiparib 60 mg BID was administered continuously and TMZ 60 mg was pulsed for Days 1 through 7 of the 28‐day cycle. The median pamiparib treatment duration was 2.30 months (range, 0.2–23.9 months) and the median pamiparib dose intensity per patient was 110.6 mg/day (range, 34.3–120.4 mg/day).

### 3.2 Safety and Tolerability

In the dose‐escalation stage, all patients (100%) experienced at least one TEAE (Table 2) and 57 patients (86.4%) experienced a TEAE considered to be related to pamiparib or TMZ (Table S1). Four patients (6.1%) discontinued any study medication due to a TEAE (Table 2). The most common TEAEs of any grade were anemia (37 patients, 56.1%), nausea (36 patients, 54.5%), and fatigue (32 patients, 48.5%). These were also the most common treatment‐related TEAEs to either pamiparib or TMZ (anemia: 36 patients, 54.5%; nausea: 27 patients, 40.9%; fatigue: 25 patients, 37.9%) (Table S1). Fifty patients (75.8%) experienced Grade 3 or higher TEAEs (Table 2), the most common of which were anemia (23 patients, 34.8%), neutropenia (18 patients, 27.3%), thrombocytopenia (13 patients, 19.7%), and neutrophil count decreased (11 patients, 16.7%). Twenty patients (30.3%) experienced serious TEAEs, the most common of which were abdominal pain (3 patients, 4.5%) and pneumonia (2 patients, 3.0%). There were 44 patients (66.7%) with one or more TEAEs leading to dose reduction or interruption. There were no reported cases of myelodysplastic syndrome nor acute myeloid leukemia.

**TABLE 2:** Summary of AEs and the most common TEAEs by preferred term (safety population).

	Dose escalation									Dose expansion^a^					
	Arm A: Pulsed						Arm B: Continuous		Total						
*n* (%)	PAM 60 mg + TMZ 40 mg 7‐day pulse (*n* = 4)	PAM 60 mg + TMZ 60 mg 7‐day pulse (*n* = 13)	PAM 60 mg + TMZ 80 mg 7‐day pulse (*n* = 9)	PAM 60 mg + TMZ 100 mg 7‐day pulse (*n* = 3)	PAM 60 mg + TMZ 120 mg 7‐day pulse (*n* = 3)	PAM 60 mg + TMZ 40 mg 14‐day pulse (*n* = 14)	PAM 60 mg + TMZ 20 mg cont (*n* = 14)	PAM 60 mg + TMZ 40 mg + cont (*n* = 6)	All patients (*n* = 66)	Cohort 1: OC *BRCA*/HRD+ (*n* = 4)	Cohort 2: TNBC *BRCA*/HRD+ (*n* = 1)	Cohort 4: SCLC (*n* = 22)	Cohort 5: gastric (*n* = 21)	Cohort 6: other HRD+ (*n* = 25)	All patients (*n* = 73)
Patients with ≥1 TEAE	4 (100)	13 (100)	9 (100)	3 (100)	3 (100)	14 (100)	14 (100)	6 (100)	66 (100)	4 (100)	1 (100)	22 (100)	20 (95.2)	24 (96.0)	71 (97.3)
Grade 3 or higher TEAE	2 (50.0)	10 (76.9)	6 (66.7)	2 (66.7)	3 (100)	13 (92.9)	10 (71.4)	4 (66.7)	50 (75.8)	4 (100)	1 (100)	18 (81.8)	16 (76.2)	16 (64.0)	55 (75.3)
Serious TEAE	1 (25.0)	4 (30.8)	1 (11.1)	2 (66.7)	2 (66.7)	5 (35.7)	5 (35.7)	0	20 (30.3)	3 (75.0)	0	9 (40.9)	9 (42.9)	9 (36.0)	30 (41.1)
TEAE leading to death	0	0	0	0	0	1 (7.1)	0	0	1 (1.5)	0	0	1 (4.5)	1 (4.8)	0	2 (2.7)
TEAE leading to treatment discontinuation															
PAM only	0	0	0	0	1 (33.3)	0	0	0	1 (1.5)	0	0	0	0	0	0
TMZ only	0	0	0	0	0	0	0	0	0	1 (25.0)	0	0	0	0	1 (1.4)
PAM and TMZ	0	0	0	1 (33.3)	1 (33.3)	1 (7.1)	1 (7.1)	0	4 (6.1)	1 (25.0)	0	1 (4.5)	1 (4.8)	2 (8.0)	5 (6.8)
PAM or TMZ	0	0	0	1 (33.3)	1 (33.3)	1 (7.1)	1 (7.1)	0	4 (6.1)	2 (50.0)	0	1 (4.5)	1 (4.8)	2 (8.0)	6 (8.2)
Most common TEAEs by preferred term (≥20% of total)^b^															
Anemia	0	8 (61.5)	5 (55.6)	2 (66.7)	3 (100)	10 (71.4)	6 (42.9)	3 (50.0)	37 (56.1)	3 (75.0)	1 (100)	13 (59.1)	13 (61.9)	16 (64.0)	46 (63.0)
Nausea	3 (75.0)	9 (69.2)	5 (55.6)	2 (66.7)	2 (66.7)	5 (35.7)	6 (42.9)	4 (66.7)	36 (54.5)	4 (100)	1 (100)	12 (54.5)	12 (57.1)	7 (28.0)	36 (49.3)
Fatigue	2 (50.0)	9 (69.2)	4 (44.4)	1 (33.3)	0	10 (71.4)	4 (28.6)	2 (33.3)	32 (48.5)	3 (75.0)	0	13 (59.1)	12 (57.1)	7 (28.0)	35 (47.9)
Thrombocytopenia	1 (25.0)	5 (38.5)	4 (44.4)	1 (33.3)	3 (100)	7 (50.0)	1 (7.1)	2 (33.3)	24 (36.4)	2 (50.0)	0	7 (31.8)	6 (28.6)	4 (16.0)	19 (26.0)
Neutropenia^c^	1 (25.0)	4 (30.8)	3 (33.3)	2 (66.7)	3 (100)	5 (35.7)	3 (21.4)	1 (16.7)	22 (33.3)	4 (100)	1 (100)	7 (31.8)	5 (23.8)	5 (20.0)	22 (30.1)
Decreased appetite	1 (25.0)	6 (46.2)	2 (22.2)	1 (33.3)	0	4 (28.6)	5 (35.7)	1 (16.7)	20 (30.3)	3 (75.0)	0	11 (50.0)	8 (38.1)	4 (16.0)	26 (35.6)
Diarrhea	0	3 (23.1)	3 (33.3)	1 (33.3)	1 (33.3)	4 (28.6)	3 (21.4)	2 (33.3)	17 (25.8)	2 (50.0)	1 (100.0)	3 (13.6)	5 (23.8)	3 (12.0)	14 (19.2)
Vomiting	1 (25.0)	5 (38.5)	1 (11.1)	1 (33.3)	2 (66.7)	4 (28.6)	2 (14.3)	1 (16.7)	17 (25.8)	2 (50.0)	0	7 (31.8)	10 (47.6)	4 (16.0)	23 (31.5)
Platelet count decreased	1 (25.0)	0	3 (33.3)	1 (33.3)	0	2 (14.3)	3 (21.4)	2 (33.3)	12 (18.2)	1 (25.0)	1 (100)	9 (40.9)	5 (23.8)	8 (32.0)	24 (32.9)
Neutrophil count decreased^c^	0	2 (15.4)	2 (22.2)	1 (33.3)	0	2 (14.3)	3 (21.4)	2 (33.3)	12 (18.2)	0	0	7 (31.8)	4 (19.0)	8 (32.0)	19 (26.0)
Constipation	0	1 (7.7)	0	0	0	1 (7.1)	5 (35.7)	1 (16.7)	8 (12.1)	2 (50.0)	0	7 (31.8)	5 (23.8)	4 (16.0)	18 (24.7)

Abbreviations: *BRCA*, breast cancer susceptibility gene; HRD+, homologous recombination deficiency; OC, ovarian cancer; PAM, pamiparib; SCLC, small cell lung cancer; TEAE, treatment‐emergent adverse event; TMZ, temozolomide; TNBC, triple‐negative breast cancer.

^a^ No patients were enrolled in cohort 3 (metastatic castration‐resistant prostate cancer).

^b^ TEAEs occurring in either the dose‐escalation or dose‐expansion stage in ≥20% of total patients.

^c^ Diagnosis of and preferred term selection of neutropenia or neutrophil count decreased was at the investigator's discretion.

In the dose‐escalation stage, four patients experienced DLTs, two patients each in the TMZ 100‐mg group (neutropenia and neutrophil count decreased) and TMZ 120‐mg group (both neutropenia) (Table S2). All four DLTs were Grade 4 TEAEs considered to be related to pamiparib and TMZ; none were serious. One patient in the 40‐mg TMZ 14‐day pulsed group experienced a TEAE of biliary sepsis leading to death; this TEAE was not considered to be related to study drug treatment. The MTD was 60 mg pamiparib BID with 7‐day pulsed TMZ 60 mg QD or continuous TMZ 20 mg QD. Based on an overall safety assessment with lower incidences of thrombocytopenia, neutropenia, decreased platelet count, and decreased neutrophil count, the RP2D administered in the dose‐expansion stage was chosen to be 60 mg pamiparib BID with 7‐day pulsed TMZ 60 mg QD.

In the dose‐expansion stage, 71 patients (97.3%) experienced at least one TEAE (Table 2) and 66 patients (90.4%) experienced a TEAE considered to be related to pamiparib or TMZ (Table S1). Six patients (8.2%) discontinued any study medication due to a TEAE (Table 2). As in the dose‐escalation stage, the most common TEAEs of any grade were anemia (46 patients, 63.0%), nausea (36 patients, 49.3%), and fatigue (35 patients, 47.9%). These were also the most common TEAEs that were considered treatment‐related to either pamiparib or TMZ (anemia: 43 patients, 58.9%; nausea: 31 patients, 42.5%; fatigue: 25 patients, 34.2%) (Table S1). Fifty‐five patients (75.3%) experienced a Grade 3 or higher TEAE, the most common of which were anemia (26 patients, 35.6%), neutropenia (16 patients, 21.9%), neutrophil count decreased (15 patients, 20.5%), and thrombocytopenia (13 patients, 17.8%). Thirty patients (41.1%) experienced serious TEAEs, the most common of which were anemia (4 patients, 5.5%), abdominal pain (3 patients, 4.1%), neutropenia (3 patients, 4.1%), and thrombocytopenia (3 patients, 4.1%). There were 51 patients (69.9%) with at least one TEAE leading to dose reduction or interruption. One patient each experienced a TEAE leading to death in the gastric cancer cohort (cohort 5; upper gastrointestinal bleeding) and small cell lung cancer (SCLC) cohort (cohort 4; respiratory insufficiency); neither TEAE was considered to be related to study drug treatment.

### 3.3 Antitumor Activity

**FIGURE 2:**
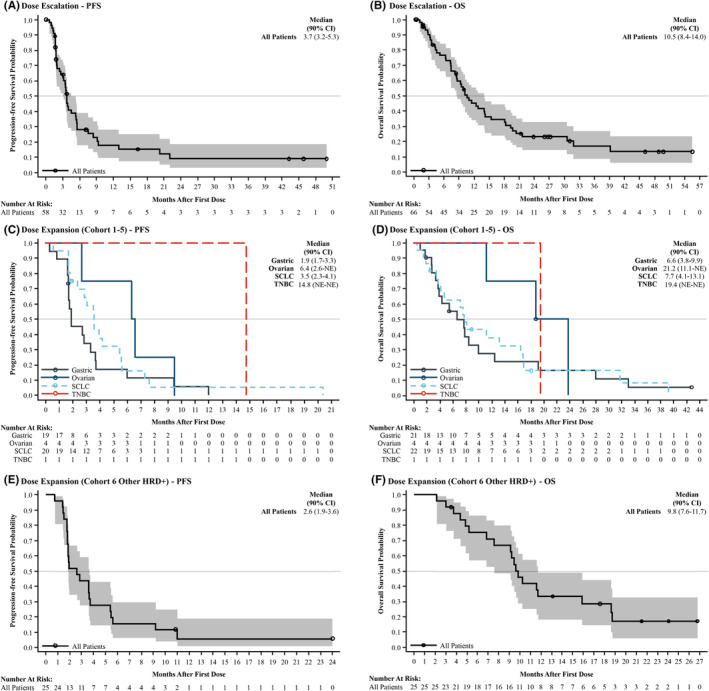
Progression‐free survival (PFS) and overall survival (OS) in the efficacy‐evaluable population. Kaplan‐Meier plots of (A) PFS and (B) OS in the dose‐escalation stage. Kaplan‐Meier plots of (C) PFS and (D) OS by tumor type in cohorts 1‐5 of the dose‐expansion stage. Kaplan‐Meier plots of (E) PFS and (F) OS in cohort 6 of the dose‐expansion stage. No patients were enrolled in cohort 3 (metastatic castration‐resistant prostate cancer) of the dose‐expansion stage. CI, confidence interval; HRD+, homologous recombination deficiency; NE, not estimable; OS, overall survival; PFS, progression‐free survival; SCLC, small cell lung cancer; TNBC, triple‐negative breast cancer.

In the dose‐escalation, efficacy‐evaluable population, the confirmed ORR (90% CI) was 13.8% (7.1%–23.5%) (Table 3). No patients experienced confirmed CR, eight patients (13.8%) achieved PR, and 29 patients (50.0%) had SD (Table 3). The median (90% CI) DoR was 7.7 months (3.7–13.0 months) and the DCR (90% CI) was 63.8% (52.2%–74.3%). The median PFS (90% CI) was 3.7 months (3.2–5.3 months) and median OS (90% CI) in the safety population was 10.5 months (8.4–14.0 months) (Table 3; Figure 2A,B).

**TABLE 3:** Summary of clinical activity (efficacy‐evaluable population).

	Dose escalation									Dose expansion^a^					
	Arm A: Pulsed						Arm B: Continuous		Total						
	PAM 60 mg + TMZ 40 mg 7‐day pulse (*n* = 3)	PAM 60 mg + TMZ 60 mg 7‐day pulse (*n* = 12)	PAM 60 mg + TMZ 80 mg 7‐day pulse (*n* = 8)	PAM 60 mg + TMZ 100 mg 7‐day pulse (*n* = 2)	PAM 60 mg + TMZ 120 mg 7‐day pulse (*n* = 2)	PAM 60 mg + TMZ 40 mg 14‐day pulse (*n* = 14)	PAM 60 mg + TMZ 20 mg cont (*n* = 13)	PAM 60 mg + TMZ 40 mg cont (*n* = 4)	All patients (*n* = 58)	Cohort 1: OC *BRCA*/HRD+ (*n* = 4)	Cohort 2: TNBC *BRCA*/HRD+ (*n* = 1)	Cohort 4: SCLC (*n* = 20)	Cohort 5: gastric (*n* = 19)	Cohort 6: other HRD+ (*n* = 25)	All patients (*n* = 69)
Best response, *n* (%)															
CR	0	0	0	0	0	0	0	0	0	0	0	0	0	1 (4.0)	1 (1.4)
PR	0	2 (16.7)	2 (25.0)	0	0	3 (21.4)	0	1 (25.0)	8 (13.8)	2 (50.0)	1 (100)	3 (15.0)	0	1 (4.0)	7 (10.1)
SD	1 (33.3)	9 (75.0)	4 (50.0)	2 (100)	1 (50.0)	3 (21.4)	7 (53.8)	2 (50.0)	29 (50.0)	1 (25.0)	0	12 (60.0)	8 (42.1)	11 (44.0)	32 (46.4)
PD	2 (66.7)	1 (8.3)	2 (25.0)	0	1 (50.0)	7 (50.0)	4 (30.8)	0	17 (29.3)	1 (25.0)	0	4 (20.0)	10 (52.6)	12 (48.0)	27 (39.1)
Could not be determined	0	0	0	0	0	1 (7.1)	2 (15.4)	1 (25.0)	4 (6.9)	0	0	1 (5.0)	1 (5.3)	0	2 (2.9)
ORR, *n* (%)	0	2 (16.7)	2 (25.0)	0	0	3 (21.4)	0	1 (25.0)	8 (13.8)	2 (50.0)	1 (100.0)	3 (15.0)	0	2 (8.0)	8 (11.6)
Median PFS (90% CI), months	2.0 (1.2‐NE)	5.6 (2.7–22.0)	5.3 (1.8–7.5)	NE (4.5‐NE)	NE (1.8‐NE)	2.8 (1.7–3.7)	3.1 (1.7–3.9)	3.5 (1.1‐NE)	3.7 (3.2–5.3)	6.4 (2.6‐NE)	14.8 (NE‐ NE)	3.5 (2.3–4.1)	1.9 (1.7–3.3)	2.6 (1.9–3.6)	2.8 (1.9–3.6)
Median OS (90% CI), months^b^	7.6 (2.2‐NE)	14.8 (8.4‐NE)	12.7 (9.4‐NE)	12.4 (4.5‐NE)	12.3 (4.2‐NE)	6.3 (3.4–31.8)	13.9 (8.0‐NE)	8.2 (1.1–12.7)	10.5 (8.4–14.0)	21.2 (11.1‐NE)	19.4 (NE‐ NE)	7.7 (4.1–13.1)	6.6 (3.8–9.9)	9.8 (7.6–11.7)	9.2 (7.6–11.1)

Abbreviations: *BRCA*, breast cancer susceptibility gene; cont, continuous; CR, complete response; NE, not estimable; ORR, objective response rate; OC, ovarian cancer; OS, overall survival; PAM, pamiparib; PR, partial response; PFS, progression‐free survival; PD, progressive disease; SCLC, small cell lung cancer; SD, stable disease; TMZ, temozolomide; TNBC, triple‐negative breast cancer.

^a^ No patients were enrolled in cohort 3 (metastatic castration‐resistant prostate cancer).

^b^ OS evaluated in the safety population.

In the dose‐expansion stage efficacy‐evaluable population, the confirmed ORR (90% CI) was 11.6% (5.9%–19.9%). One patient (1.4%) with leiomyosarcoma that was HRD+ and had a germline *BRCA2* mutation experienced confirmed CR, seven patients (10.1%) achieved PR, and 32 patients (46.4%) had SD. The median (90% CI) DoR was 5.7 months (3.8–11.0 months) and the DCR (90% CI) was 58.0% (47.4%–68.1%). Median PFS (90% CI) was 2.8 months (1.9–3.6 months), and median OS (90% CI) in the safety population was 9.2 months (7.6–11.1 months) (Table 3; Figure 2C–F). The highest median OS (90% CI) was 9.8 months (7.6–11.1 months) in the other HRD+ group (cohort 6), and the highest median PFS (90% CI) was 3.5 months (2.3–4.1 months) in the SCLC group (cohort 4), excluding the ovarian cancer (*n* = 4; cohort 1) and TNBC (*n* = 1; cohort 2) groups due to small sample sizes (Table 3).

**FIGURE 3:**
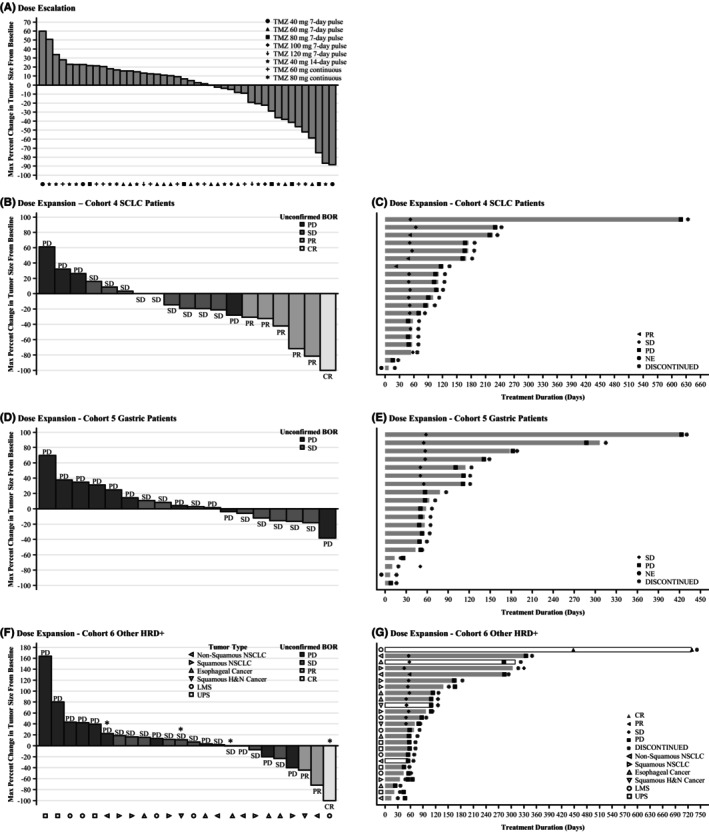
Best percent change from baseline in target lesion–best overall response (efficacy‐evaluable population). (A) Shows patients with measurable disease at baseline and postbaseline, excluding prostate cancer patients. (B, D, and F) Show unconfirmed best overall response in patients with measurable disease at baseline and postbaseline. (C, E, and G) Show confirmed best overall and/or last response. In cohort 6, the four patients with a *BRCA1/2* mutation are indicated with an asterisk (panel F) or open rectangles (panel G). BOR, best overall response; CR, complete response; EC, esophageal cancer; HRD+, homologous recombination deficiency; LMS, leiomyosarcoma; NE, not estimable; NSCLC, non‐small cell lung cancer; NSN, non‐squamous NSCLC; PD, progressive disease; PR, partial response; SD, stable disease; SCLC, small cell lung cancer; SHN, squamous head and neck cancer; SN, squamous NSCLC; TMZ, temozolomide; UPS, undifferentiated pleomorphic sarcoma.

The confirmed ORR for both the dose‐escalation and ‐expansion stages combined was 12.6% (16/127 patients). The maximum percent change from baseline in tumor size for both stages is shown in Figure 3.

An exploratory biomarker analysis included 46 efficacy evaluable patients with known GIS score and optional tumor *BRCA* mutation status from the dose‐escalation phase and cohorts 1–5 of the dose‐expansion phase. Patients with GIS ≥33 (*n* = 13) showed better efficacy compared with GIS <33 patients (*n* = 33) regardless of the tumor *BRCA* mutation status (Tables S3 and S4). The confirmed ORR (90% CI) was 46.2% (22.4%–71.3%) in GIS ≥33 patients compared with 9.1% (2.5%–21.9%) in GIS <33 patients (Table S3). The confirmed DCR (90% CI) was 92.3% (68.4%–99.6%) in GIS ≥33 patients compared with 54.5% (38.9%–69.5%) in GIS <33 patients (Table S4). However, in the 25 additional patients enrolled in cohort 6 of the dose‐expansion phase (HRD+ defined as GIS ≥33, mixed solid tumors) for further efficacy confirmation, the confirmed ORR (90% CI) was 8.0% (1.4%–23.1%) and the DCR (90% CI) was 52% (34.1%–69.5%) (Table 3).

### 3.4 Pharmacokinetics

**FIGURE 4:**
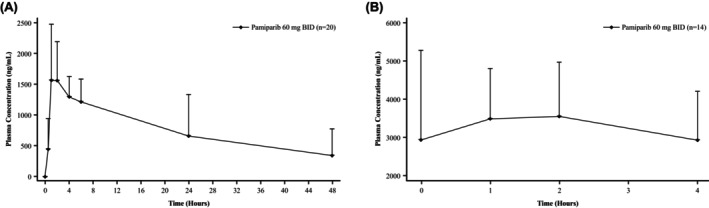
Pamiparib plasma concentration‐time profiles. (A) Cycle 1 Day ‐2. (B) Cycle 1 Day 15. Shown are the arithmetic mean + standard deviation from blood samples collected from patients at various timepoints following administration of pamiparib. BID, twice daily.

After steady state was reached with combination TMZ therapy (cycle 1 Day 15), the pamiparib geometric mean AUC_0‐4_ was 11,119 h × ng/mL. A summary of pamiparib PK parameters is provided in Table S5. For patients who received combination pamiparib and TMZ from cycle 1 Day 1, geometric mean pamiparib concentration values at 2 h post dose on cycle 1 Day 1 (1577 ng/mL) and cycle 1 Day 15 (3480 ng/mL) were consistent with its single‐agent values (Figure 4). The mean plasma concentration of TMZ 1 h after oral administration on cycle 1 Day 1 increased near proportionally with dose, ranging from 338 ng/mL (20‐mg dose) to 1510 ng/mL (120‐mg dose), also consistent with its single‐agent values.

## 4 DISCUSSION

This phase 1b open‐label, multicenter study investigated the safety, tolerability, and clinical activity of oral pamiparib in combination with TMZ in patients with locally advanced and metastatic solid tumors. In the dose‐escalation stage, continuous pamiparib 60 mg in combination with various dosing regimens of TMZ were investigated; the MTD of TMZ was 7‐day pulsed 60 mg QD or continuous 20 mg QD. The 7‐day pulsed 60‐mg dose was chosen as the RP2D for the dose‐expansion stage based on the lack of observed drug–drug interactions, the DCR across dosing cohorts, and the safety profile from the escalation stage, including the DLTs experienced at higher doses of TMZ.

In the dose‐escalation stage, four DLTs were observed at TMZ doses of 100 and 120 mg, all of which were hematologic (three neutropenia, one neutrophil count decreased). Neutropenia was among the most common Grade 3 or higher TEAEs in the dose‐escalation and dose‐expansion stages (27.3% and 21.9%, respectively), along with other hematologic TEAEs of anemia (34.8% and 35.6%), thrombocytopenia (19.7% and 17.8%), and neutrophil count decreased (16.7% and 20.5%). This was expected as hematologic toxicities are commonly reported with PARP inhibitor monotherapy^29^ and myelosuppression has occurred with TMZ therapy, with higher risk of myelosuppression in women and older patients.^11^ Hematologic events have been the most common Grade 3 or higher TEAEs in prior studies of pamiparib monotherapy in clinical trials of advanced or metastatic solid tumors (2.5–160 mg BID or QD)^24^ and TNBC or hormone receptor‐positive breast cancer (60 mg BID).^26^ In prior combination trials of PARP inhibitors with TMZ, hematologic AEs were also common for veliparib in small cell lung cancer or metastatic melanoma,^19^, ^21^ olaparib in recurrent glioblastoma,^14^ and talazoparib in advanced malignancies.^16^ Rates of Grade 3 or higher neutropenia and thrombocytopenia observed in both stages of this study were similar to Grade 3/4 events with combination TMZ and veliparib (16%–31% and 20%–50%, respectively),^19^, ^21^ olaparib (13% and 25%),^14^ or talazoparib (related AEs, 28% and 33%).^16^

The ORR in the dose‐escalation (8/58, 13.8%) and dose‐expansion stages (8/69, 11.6%) were similar. In the dose‐expansion stages, no patients achieved an objective response to treatment in the gastric cancer group. The highest ORR was reported in those with SCLC (3/20, 15%) (excluding ovarian cancer [cohort 1] and TNBC [cohort 2] due to small sample sizes). Two of the four patients with ovarian cancer (cohort 1) in the dose‐expansion stage achieved PR, which was comparable to the efficacy observed for pamiparib monotherapy in ovarian cancer that was platinum sensitive (ORR, 64.6%) or platinum resistant (ORR, 31.6%).^25^ Compared with the combination of veliparib and TMZ in two studies, the ORR in this study for both stages combined (12.6%, 16/127) was slightly higher than that reported in patients with metastatic melanoma (8.7%–10.3%)^19^ but lower than in patients with SCLC (39.0%).^21^ HRD status was not required for inclusion in either of these two previously reported studies,^19^, ^21^ and both studies reported improved survival in subgroups of patients with DNA repair gene mutations.^19^, ^21^ The median PFS and OS in the dose‐escalation (3.7 and 10.5 months, respectively) and dose‐expansion (2.8 and 9.2 months) stages were similar to ranges reported for veliparib combined with TMZ in SCLC or metastatic melanoma (median PFS, 3.6–3.8 months; median OS, 8.2–13.6 months).^19^, ^21^ Patients in this study were heavily pretreated prior to enrollment, which may have contributed to the modest antitumor activity.

This study enrolled a patient population with tumors likely to harbor DNA damage repair deficiencies such as *BRCA1/2* gene mutations and/or HRD that were particularly sensitive to PARP inhibition due to synthetic lethality. Patients in dose‐expansion cohort 1 (ovarian cancer) or cohort 2 (TNBC) were required to have historical or central *BRCA1/2* mutation or HRD+ for enrollment. An exploratory biomarker analysis of efficacy evaluable patients with known GIS score and optional tumor *BRCA* mutation status from the dose‐escalation phase and cohorts 1–5 of the dose‐expansion phase showed that patients with GIS ≥33 had superior ORR and DCR compared with GIS <33 patients regardless of tumor *BRCA* mutation status. Therefore, GIS ≥33 was selected as the cutoff for HRD+ in the additional patients enrolled in cohort 6 with other HRD+ solid tumor types of the dose‐expansion phase to further investigate the efficacy of pamiparib plus TMZ; a response of 8% was observed in these patients, which was much lower than the previously observed response of 46.2% in the patients with GIS ≥33 of the aforementioned exploratory biomarker analysis. However, it is noteworthy that the tumor types varied considerably in these two populations, with nine of the 13 patients of GIS ≥33 in the exploratory biomarker analysis having tumor types known to be sensitive to PARP inhibitor (i.e., ovarian cancer, prostate cancer, or breast cancer). However, in cohort 6, one out of four enrolled patients with non‐squamous NSCLC achieved PR (25%) and one out of five enrolled patients with leiomyosarcoma achieved CR (20%). Interestingly, one patient with leiomyosarcoma with GIS ≥33 in the exploratory biomarker analysis also demonstrated PR, echoing a recently published study showing response to olaparib monotherapy in patients with HRD+ uterine leiomyosarcoma (defined by targeted panel testing, whole exome sequencing, or whole genome sequencing).^30^ These results indicate that HRD status could potentially predict the efficacy of pamiparib in some tumor types, such as non‐squamous NSCLC and leiomyosarcoma. The role of HRD+ status as a potential biomarker for pamiparib, with and without TMZ, needs to be further evaluated in trials with larger sample sizes.

Administration of pamiparib in combination with TMZ did not notably impact the PK profile of pamiparib. Geometric mean AUC_0‐4_ at steady state was approximately twice the value after single‐dose pamiparib administration, which is consistent with its half‐life, suggesting a lack of drug–drug interaction between pamiparib and TMZ. For patients who received pamiparib and TMZ combination from cycle 1 Day 1, their concentration values at 2 h postdose on cycle 1 Day 1 and cycle 1 Day 15 were also consistent with the respective single‐agent values, also suggesting a lack of drug–drug interaction. The near‐proportional dose increase in plasma TMZ concentration was consistent with its single‐agent values, which suggests lack of drug–drug interaction between the two agents, likely due to the non‐overlapping metabolic pathway of respective drug.

The results of this study were impacted by several limitations. As this was a phase 1b trial including a wide range of malignancies, there was a small sample size for each tumor type and sample sizes between groups varied greatly from one to 25 patients. Sample sizes were especially small for ovarian cancer (cohort 1) and TNBC (cohort 2), as enrollment was stopped early due to difficulties in recruitment. Additionally, this study enrolled predominantly White patients and had little racial and ethnic diversity. Historical HRD status was not routinely available for tumor types not classically associated with HRD mutations and, as such, identifying HRD+ status in these tumor types was dependent on tissue availability. Screening for HRD+ patients was generally time consuming and may have been biased toward enrolling patients with slower growing tumor types. Pamiparib monotherapy has previously demonstrated durable response in ovarian cancers,^25^ making it difficult to comment on the additive effect of pamiparib with TMZ combination therapy in this cancer type.

In this study, pamiparib in combination with TMZ had a manageable safety profile. However, the efficacy of this combination in specific tumor types regarding HRD and/or *BRCA* mutation status needs further exploration in larger trials with sufficient patient numbers for each tumor type. The combination of pamiparib plus TMZ may be most effective in patients with tumor types known to be sensitive to PARP inhibitors (i.e., ovarian cancer, prostate cancer, or breast cancer) with HRD+ status determined using a GIS cutoff of ≥33. The optimal GIS cutoff may need to be determined for other tumor types. Future studies will need to enroll both treatment‐naïve and previously treated patients with a given tumor type in order to determine the impact of prior treatment on efficacy outcomes with pamiparib and TMZ.

## AUTHOR CONTRIBUTIONS

**Agostina Stradella**: Data curation (equal), methodology (equal), project administration (equal), resources (equal), writing – drafting (equal), writing – review and editing (equal). **Melissa Johnson**: Data curation (equal), methodology (equal), project administration (equal), resources (equal), writing – drafting (equal), writing – review and editing (equal). **Sanjay Goel**: Data curation (equal), methodology (equal), project administration (equal), resources (equal), writing – drafting (equal), writing – review and editing (equal). **Haeseong Park**: Data curation (equal), methodology (equal), project administration (equal), resources (equal), writing – drafting (equal), writing – review and editing (equal). **Nehal Lakhani**: Data curation (equal), methodology (equal), project administration (equal), resources (equal), writing – drafting (equal), writing – review and editing (equal). **Hendrik‐Tobias Arkenau**: Data curation (equal), methodology (equal), project administration (equal), resources (equal), writing – drafting (equal), writing – review and editing (equal). **Matthew D. Galsky**: Data curation (equal), methodology (equal), project administration (equal), resources (equal), writing – drafting (equal), writing – review and editing (equal). **Emiliano Calvo**: Data curation (equal), methodology (equal), project administration (equal), resources (equal), writing – drafting (equal), writing – review and editing (equal). **Vicente Baz**: Data curation (equal), methodology (equal), project administration (equal), resources (equal), writing – drafting (equal), writing – review and editing (equal). **Victor Moreno**: Data curation (equal), methodology (equal), project administration (equal), resources (equal), writing – drafting (equal), writing – review and editing (equal). **Omar Saavedra**: Data curation (equal), methodology (equal), project administration (equal), resources (equal), writing – drafting (equal), writing – review and editing (equal). **Stephen J. Luen**: Data curation (equal), methodology (equal), project administration (equal), resources (equal), writing – drafting (equal), writing – review and editing (equal). **Song Mu**: Formal analysis (equal), methodology (equal), writing – drafting (equal), writing – review and editing (equal). **Qiting Wan**: Formal analysis (equal), methodology (equal), writing – drafting (equal), writing – review and editing (equal). **Victoria Chang**: Formal analysis (equal), methodology (equal), writing – drafting (equal), writing – review and editing (equal). **Wa Zhang**: Formal analysis (equal), methodology (equal), writing – drafting (equal), writing – review and editing (equal). **Minal Barve**: Conceptualization (lead), data curation (equal), formal analysis (equal), methodology (equal), project administration (equal), resources (equal), writing – drafting (equal), writing – review and editing (equal).

## FUNDING INFORMATION

This study was funded by BeiGene, Ltd.

## CONFLICT OF INTEREST STATEMENT

Sanjay Goel and Minal Barve have no conflicts of interest to report. Stephen J. Luen has received research funding to his institution from AstraZeneca, BeiGene, Novartis, Roche, and SpringWorks Therapeutics. Agostina Stradella has been a paid participant in a speaker's bureau for Daiichi and Novartis and has had travel, accommodations or expenses paid by Eisai, Novartis, and Pfizer, and has consulted or served an advisory role for AstraZeneca, Boehringer Ingelheim, Novartis, and Seagen. Melissa Johnson has received research funding to the institution from AbbVie, Acerta, Adaptimmune, Amgen, Apexigen, Arcus Biosciences, Array BioPharma, Artios Pharma, AstraZeneca, Atreca, BeiGene, BerGenBio, BioAtla, Black Diamond, Boehringer Ingelheim, Bristol Myers Squibb, Calithera Biosciences, Carisma Therapeutics, Checkpoint Therapeutics, City of Hope National Medical Center, Corvus Pharmaceuticals, Curis, CytomX, Daiichi Sankyo, Dracen Pharmaceuticals, Dynavax, Lilly, Elicio Therapeutics, EMD Serono, EQRx, Erasca, Exelixis, Fate Therapeutics, Genentech/Roche, Genmab, Genocea Biosciences, GlaxoSmithKline, Gritstone Oncology, Guardant Health, Harpoon, Helsinn Healthcare SA, Hengrui Therapeutics, Hutchison MediPharma, IDEAYA Biosciences, IGM Biosciences, Immunitas Therapeutics, Immunocore, Incyte, Janssen, Jounce Therapeutics, Kadmon Pharmaceuticals, Kartos Therapeutics, Loxo Oncology, Lycera, Memorial Sloan Kettering Cancer Center, Merck, Merus, Mirati Therapeutics, Mythic Therapeutics, NeoImmune Tech, Neovia Oncology, Novartis, Numab Therapeutics, Nuvalent, OncoMed Pharmaceuticals, Palleon Pharmaceuticals, Pfizer, PMV Pharmaceuticals, Rain Therapeutics, RasCal Therapeutics, Regeneron Pharmaceuticals, Relay Therapeutics, Revolution Medicines, Ribon Therapeutics, Rubius Therapeutics, Sanofi, Seven and Eight Biopharmaceuticals / Birdie Biopharmaceuticals, Shattuck Labs, Silicon Therapeutics, Stem CentRx, Syndax Pharmaceuticals, Takeda Pharmaceuticals, Tarveda, TCR2 Therapeutics, Tempest Therapeutics, Tizona Therapeutics, TMUNITY Therapeutics, Turning Point Therapeutics, University of Michigan, Vyriad, WindMIL Therapeutics, and Y‐mAbs Therapeutics; and has consulted or served an advisory role for AbbVie, Amgen, Arcus Biosciences, Arrivent, Astellas, AstraZeneca, Black Diamond, Boehringer Ingelheim, Calithera Biosciences, Daiichi Sankyo, EcoR1, Genentech / Roche, Genmab, Genocea Biosciences, Gilead Sciences, GlaxoSmithKline, Gritstone Oncology, Ideaya Biosciences, Immunocore, iTeos, Janssen, Jazz Pharmaceuticals, Merck, Mirati Therapeutics, Molecular Axiom, Normunity, Novartis, Oncorus, Pyramid Biosciences, Regeneron Pharmaceuticals, Revolution Medicines, Sanofi‐Aventis, SeaGen, Synthekine, Takeda Pharmaceuticals, Turning Point Therapeutics, and VBL Therapeutics. Haeseong Park has received researching funding to the institution from Adlai Nortye USA, Ambrx, Aprea Therapeutics AB, Array BioPharma, AstraZeneca, BJ Bioscience, Bristol Myers Squibb, Daiichi Pharmaceutical, Elicio Therapeutics, Exelixis, Fate Therapeutics, Genentech, GlaxoSmithKline, Gossamer Bio, Hutchison MediPharma, ImmuneOncia Therapeutics, ImmunoGen, Mabspace Biosciences, MacroGenics, Merck, Mersana therapeutics, Mirati Therapeutics, Novartis Pharmaceuticals, Oncologie, PsiOxus Therapeutics, RePare Therapeutics, Seattle Genetics, Synermore Biologics, TopAlliance Biosciences, Tizona, Turning Point Therapeutics, Vedanta Biosciences, and Xencor. Nehal Lakhani has received research funding to the institution from Alexo Therapeutics, Alkermes, Alpine Biosciences, Alpine Immune Sciences, Apexian Pharmaceuticals, Ascentage Pharma, Astellas Pharma, BeiGene, Biosplice/Samumed, Celgene, Constellation Pharmaceuticals, Coordination Therapeutics, CytomX Therapeutics, Epizyme, Forty Seven, Genmab, Gilead, Helsinn Therapeutics, GlaxoSmithKline, Ikena, Ikena Oncology, Incyte, InhibRx, Innovent Biologics, Jounce Therapeutics, KSQ, LAM Therapeutics, Lilly, Livzon, Loxo, Macrogenics, Merck, Mersana, Northern Biologics, Odonate, Pfizer, Regeneron, Repare Therapeutics, Sapience Therapeutics, Seagen, Servier, Shattuck Labs, SK Life Sciences, Symphogen, Tesaro, and Tizona; and has consulted or served an advisory role for S.K. Life Sciences and Ikena Oncology. Hendrik‐Tobias Arkenau has consulted or served an advisory role for BeiGene. Matthew D. Galsky has received research funding from AstraZeneca, Bristol Myers Squibb, Dendreon, Genentech, Merck, and Novartis; Matthew D. Galsky has consulted or served an advisory role for AbbVie, Alligator, Analog Devices, Asieris, AstraZeneca, Basilea, Bristol Myers Squibb, Bicycle, Curis, Dragonfly, EMD Serono, Fujifilm, Genentech, Gilead, GlaxoSmithKline, Janssen, Merck, Numab, Pfizer, Rappta Therapeutics, SeaGen, Silverback, UroGen, and Veracyte. Emiliano Calvo owns stock or other ownership interests in Oncoart Associated and START and has received honoraria from HM Hospitales Group. Emiliano Calvo has received research funding from START and has consulted or served an advisory role directly funded for Adcendo, Amunix, Anaveon, AstraZeneca/MedImmune, Bristol Myers Squibb, Chugai Pharma, Diaccurate, Elevation Oncology, Ellipses Pharma, Genmab, Janssen‐Cilag, MonTa, MSD Oncology, Nanobiotix, Novartis, Nouscom, OncoDNA, PharmaMar, Roche/Genentech, Servier, Syneos Health, TargImmune Therapeutics, and T‐Knife. Vicente Baz has been a paid participant in a speaker's bureau for AstraZeneca, Bristol Myers Squibb, Gilead, Merck, MSD, Pfizer, Roche, and Takeda. Victor Moreno has consulted or served an advisory role directly funded for Affimed, AstraZeneca, Bayer, BMD, Janssen, Roche, and Syneos. Omar Saavedra has had travel, accommodations or expenses paid by Affimed. Song Mu, Qiting Wan, Victoria Chang, and Wa Zhang are employees of BeiGene.

## ETHICS STATEMENT

The trial was conducted in accordance with the International Council for Harmonization Good Clinical Practice, the principles of the Declaration of Helsinki, and local laws and regulations. The protocol was approved by the relevant institutional review board/independent ethics committee of each center. Prior to study activity, all patients provided written informed consent.

## Supporting information


Data S1:

## Data Availability

On request, and subject to certain criteria, conditions, and exceptions, access to individual de‐identified participant data from this study will be provided (for indications that have been approved or in programs that have been terminated). Data requests may be submitted to datadisclosure@beigene.com.
