# Supplementary material for: Phase 1b study to assess the safety, tolerability, and clinical activity of pamiparib in combination with temozolomide in patients with locally advanced or metastatic solid tumors
Source: Cancer Med. 2024 Jul 5;13(13):e7385. doi: 10.1002/cam4.7385 (PMC11226541; doi:10.1002/cam4.7385)
Supplement: Supplementary file 1 — Data S1: [file CAM4-13-e7385-s001.docx]

**Supporting Information for:**

**Phase 1b study to assess the safety, tolerability, and clinical activity of pamiparib in combination with temozolomide in patients with locally advanced or metastatic solid tumors**

Agostina Stradella,^1^ Melissa Johnson,^2^ Sanjay Goel,^3^ Haeseong Park,^4,5^ Nehal Lakhani,^6^ Hendrik-Tobias Arkenau,^7^ Matthew D. Galsky,^8^ Emiliano Calvo,^9^ Vicente Baz,^10^ Victor Moreno,^11^

Omar Saavedra,^12^ Stephen J. Luen,^13,14^ Song Mu,^15^ Qiting Wan,^16^ Victoria Chang,^17^ Wa Zhang,^16^ Minal Barve^18^

^1^ Institut Català d’Oncologia – Hospital Duran I Reynals, L’Hospitalet de Llobregat, Catalunya, Spain

^2^ Sarah Cannon Research Institute, Nashville, Tennessee, USA

^3^ Rutgers Robert Wood Johnson Medical School, New Brunswick, New Jersey, USA

^4^ Washington University School of Medicine, St. Louis, Missouri, USA

^5^ Dana-Farber Cancer Institute, Boston, Massachusetts, USA

^6^ START Midwest, Grand Rapids, Michigan, USA

^7^ HCA Healthcare, London, UK

^8^ Icahn School of Medicine at Mount Sinai, New York, New York, USA

^9^ START Madrid-CIOCC, Centro Integral Oncológico Clara Campal, Madrid, Spain

^10^ Hospital Universitario Virgen Macarena, Seville, Spain

^11^ START Madrid-FJD, Fundacion Jimenez Diaz University Hospital, Madrid, Spain

^12^ Vall d'Hebron Institute of Oncology, Barcelona, Spain

^13^ Department of Medical Oncology, Peter MacCallum Cancer Centre, Melbourne, Australia

^14^ Sir Peter MacCallum Department of Oncology, The University of Melbourne, Melbourne, Australia

^15^ BeiGene, USA Inc., Ridgefield Park, New Jersey, USA

^16^ BeiGene (Beijing) Co., Ltd., Beijing, China

^17^ BeiGene, USA Inc., San Mateo, California, USA

^18^ Mary Crowley Cancer Research, Dallas, Texas, USA

**Contents**

[Inclusion Criteria 3](#_Toc143504241)

[Exclusion Criteria 8](#_Toc143504242)

[Supporting Table 1. Summary of treatment-related adverse events and the most common treatment-related TEAEs by preferred term (safety population) 11](#_Toc143504243)

[Supporting Table 2. Dose-limiting toxicities (safety population) 14](#_Toc143504244)

[Supporting Table 3. Analysis of objective response rate (confirmed), GIS by *BRCA* (efficacy analysis set). 15](#_Toc143504245)

[Supporting Table 4. Analysis of disease control rate (confirmed), GIS by *BRCA* (efficacy analysis set) 16](#_Toc143504246)

[Supporting Table 5. Summary of pamiparib pharmacokinetic parameters 17](#_Toc143504246)

[References 18](#_Toc143504247)

# Inclusion Criteria

Patients must meet all of the following criteria to be eligible for the study:

***All Cohorts***

- Voluntarily agreed to participate by signing an informed consent
- Male or female and ≥18 years of age at the time of informed consent
- Eastern Cooperative Oncology Group performance status of ≤1
- Ability to swallow whole capsules
- Histologically or cytologically confirmed malignancy that has progressed to the advanced or metastatic stage
- Agreement to provide archival tumor tissue
  - ***Dose-Escalation Phase****:* If available, agreement to provide archival tumor tissue for exploratory biomarker analyses (Note: If archival tumor tissue is not available, an optional fresh biopsy is highly recommended)
  - ***Dose-Expansion Phase***: Patients enrolled in the homologous recombination deficiency positive (HRD+) cohorts must provide archival tissue or fresh biopsy (if archival tissue is not available) for prospective central assessment of HRD status. Other cohorts may provide tissue, if available, for retrospective analysis
- Patients must have adequate organ function as indicated by the following screening laboratory values (obtained ≤2 weeks prior to day 1):
  - Absolute neutrophil count ≥1.5 × 10^9^/L
  - Platelets ≥100 × 10^9^/L (Note: Criterion must be met without a transfusion ≤2 weeks prior to obtaining the sample)
  - Hemoglobin ≥10 g/dL or ≥6.1 mmol/L (Note: Criterion must be met without a transfusion within the 2 weeks prior to obtaining the sample)
  - Serum creatinine ≤1.5 × upper limit of normal (ULN) or estimated creatinine clearance ≥50 mL/min (calculated using the institutional standard method)
  - Total serum bilirubin ≤1.5 × ULN (total bilirubin must be <4 × ULN for patients with Gilbert’s syndrome or if indirect bilirubin concentrations are suggestive of extrahepatic source of elevation)
  - Aspartate and alanine aminotransferase ≤3 × ULN or ≤5 × ULN for patients with liver metastases
- Female patients of childbearing potential and female partners of male study patients must agree to practice highly effective methods of birth control according to The Clinical Trials Facilitation Group recommendations^1^ for the duration of the study and for ≥6 months after the last dose of study drug. In addition, non-sterile male patients must agree to practice highly effective methods of birth control^1^ and avoid sperm donation for the duration of the study and for ≥6 months after the last dose of study drug
- Willingness and ability to comply with all protocol-specified requirements

***Dose-Escalation Phase only***

- Patient must have disease that is either measurable or evaluable per Response Evaluation Criteria in Solid Tumors version (RECIST) version 1.1 criteria

***Dose Expansion Phase only***

- Patient must have measurable disease per RECIST 1.1 criteria, except where noted below (Note: tumor lesions used for freshly acquired biopsies should not be included as target lesions unless there are no other suitable target lesions available)

***Ovarian Cancer (Expansion Cohort 1)***

- Patients must have received at least one line of platinum-containing therapy in the advanced or metastatic setting
- Patients must not have progressed or have recurrent disease within 6 months after the completion of the last platinum-containing regimen (Note: Patients can receive additional therapy after the last platinum-containing regimen as long as the criteria for platinum sensitivity is met)
  - Patients with known or suspected deleterious mutations in *BRCA1* or *BRCA2* are classified as HRD+, regardless of the molecular signature result
    - If HRD or *BRCA1/2* mutation status is unknown or has not been previously evaluated, the patient must undergo tissue screening using the Myriad myChoice® HRD diagnostic test to determine eligibility

***Triple-Negative Breast Cancer (Expansion Cohort 2)***

- Patients with known or suspected deleterious mutations in *BRCA1* or *BRCA2* are classified as HRD+, regardless of the molecular signature result
  - If HRD or *BRCA1/2* mutation status is unknown or has not been previously evaluated, the patient must undergo tissue screening using the Myriad myChoice® HRD diagnostic test to determine eligibility
- No or one prior platinum-containing treatment in any treatment setting (Note: Patients could have received additional therapy after the last platinum-containing regimen as long as the other eligibility criteria are met)
- Received up to three prior lines of therapy in the advanced or metastatic setting

***Metastatic Castration-Resistant Prostate Cancer (Expansion Cohort 3)***

- Patients with known or suspected deleterious mutations in *BRCA1* or *BRCA2* are classified as HRD+, regardless of the molecular signature result
  - - If HRD or *BRCA1/2* mutation status is unknown or has not been previously evaluated, the patient must undergo tissue screening using the Myriad myChoice® HRD diagnostic test to determine eligibility
- The patient may be either chemotherapy-naïve or have previously had ≤2 taxane-based chemotherapy regimens including docetaxel and carbazitaxel; if docetaxel is used more than once, this will be considered as one regimen
- The patient may be pre- or post-treatment with a novel androgen receptor targeted agent (eg, abiraterone and/or enzalutamide)
- At least 2 weeks since the completion of prior flutamide, bicalutamide, nilutamide, or enzalutamide and abiraterone treatment
- At least 2 weeks from any radiotherapy, with the exception of a single fraction of radiotherapy for the purposes of palliation (confined to one field)
- Documented prostate cancer progression as assessed by the investigator with one of the following:
  - - Prostate-specific antigen (PSA) progression defined by a minimum of three rising PSA levels with an interval of ≥1 week between each determination. The PSA value at the screening visit should be ≥2 μg/L (2 ng/mL)
    - Radiographic progression of soft tissue disease by modified RECIST 1.1 criteria
- Surgically or medically castrated. The testosterone levels do not need to be checked as long as the patient has been on chemical castration or has undergone surgical castration for >4 months. In all cases, the luteinizing hormone-releasing hormone antagonist/agonist is to be continued in these patients
- Patients with only non-measurable bone lesions must have either progression with at least two new lesions or have PSA progression within the 6-week period before study drug administration

***Extensive-Stage Small Cell Lung Cancer (Expansion Cohort 4)***

- Received ≤2 prior lines of therapy

***Gastric/Gastroesophageal Junction Cancer (Expansion Cohort 5)***

- Received ≤2 prior lines of therapy

***HRD+ Solid Tumors, Multiple Indications (Expansion Cohort 6)***

- Patient has histologically or cytologically confirmed advanced (metastatic and/or unresectable)
  - Non-squamous non-small cell lung cancer (NSCLC)
  - Squamous NSCLC
  - Esophageal cancer
  - Squamous head and neck cancer
  - Soft-tissue sarcomas (undifferentiated pleomorphic sarcoma, leiomyosarcoma, malignant peripheral nerve sheath tumor, dedifferentiated liposarcoma, myxofibrosarcoma)
  - Patients must have tumors with HRD+ as centrally determined by the Myriad myChoice® HRD Plus assay irrespective of their known molecular signature
  - Patients with nonsquamous NSCLC, squamous NSCLC, esophageal cancer, squamous head and neck cancer must have received ≥1 but ≤3 prior lines of therapy
  - Patients with soft tissue sarcoma must have received ≥1 but ≤3 prior lines of therapy. Treatment naïve patients may be allowed if, in the opinion of the investigator, available standard-of-care first-line therapy is not appropriate

# Exclusion Criteria

Patients will be excluded from the study for any of the following reasons:

- Known hypersensitivity to any temozolomide component or to dacarbazine
- Prior treatment with a poly (ADP-ribose) polymerase (PARP) inhibitor
- Received chemotherapy, biologic therapy, immunotherapy, or investigational agent within 3 weeks prior to day 1 (or ≤5 half-lives, whichever is shorter), unless otherwise noted in the inclusion criteria
- Considered to be refractory to platinum-based therapy (eg, progressive disease at the first tumor assessment while receiving platinum treatment); for patients in the dose-expansion phase only
- Have any unresolved acute effects of any prior therapy of grade 2 or higher, except for adverse events not constituting a safety risk by investigator judgment
- Had a major surgical procedure, open biopsy, or significant traumatic injury ≤4 weeks prior to day 1, or anticipation of need for major surgical procedure during the course of the study
  - Placement of vascular access device is not considered major surgery
- Have other diagnosis of malignancy except for surgically excised non-melanoma skin cancer, adequately treated carcinoma *in situ* of the cervix, localized prostate cancer treated with curative intent, adequately treated low-stage bladder cancer, ductal carcinoma *in situ* treated surgically with curative intent, or a malignancy diagnosed >2 years ago, with no current evidence of disease and no therapy ≤2 years prior to day 1
- Has received local radiotherapy of non-target lesions for local symptom control within the last 4 weeks and must have recovered from any adverse effects of radiotherapy before recording baseline symptoms
- Have untreated leptomeningeal or brain metastasis. Patients with previously treated brain metastases are eligible if the metastases have shown no progression on brain computed tomography or magnetic resonance imaging over at least 4 weeks, the patients have no symptoms due to the brain metastases, and the patients have been off corticosteroids for ≥2 weeks
- Have active infection requiring systemic treatment
- Have known human immunodeficiency virus infection or serologic status reflecting active viral hepatitis infection as follows:
  - Patients with untreated chronic hepatitis B or chronic hepatitis B virus (HBV) carriers whose HBV DNA is >500 IU/mL should be excluded. (Note: Inactive hepatitis B surface antigen carriers and patients with treated and stable hepatitis B [HBV DNA <500 IU/mL] can be enrolled.) Patients receiving antivirals at screening should have been treated for >2 weeks before the first dose of study drug(s)
- Have any of the following cardiovascular criteria:
  - Current evidence of cardiac ischemia
  - Current symptomatic pulmonary embolism
  - Acute myocardial infarction ≤6 months prior to day 1
  - Heart failure of New York Heart Association Classification III or IV^2^ ≤6 months prior to day 1
  - Grade ≥2 ventricular arrhythmia ≤6 months prior to day 1
  - Cerebral vascular accident ≤6 months prior to day 1
- Have an active inflammatory gastrointestinal disease, chronic diarrhea, or previous complete gastric resection or lapband surgery (Note: Gastroesophageal reflux disease under treatment with proton pump inhibitors is allowed [assuming no drug interaction potential])
- Use or have anticipated need for food or drugs known to be strong or moderate cytochrome P450 (CYP)3A inhibitors or strong CYP3A inducers ≤10 days (or ≤5 half-lives, whichever is shorter) prior to day 1, including:
  - Strong CYP3A inhibitors: antibiotics (clarithromycin, telithromycin, troleandomycin), antifungals (itraconazole, ketoconazole, posaconazole, voriconazole), antivirals (boceprevir, telaprevir), protease inhibitors (indinavir, lopinavir, nelfinavir, ritonavir, saquinavir, tipranavir), and others (cobicistat, conivaptan, elvitegravir, mibefradil, nefazodone)
  - Moderate CYP3A inhibitors: antibiotics (ciprofloxacin, erythromycin), antifungals (fluconazole), protease inhibitors (amprenavir, atazanavir, darunavir, fosamprenavir), calcium channel blockers (diltiazem, verapamil), tyrosine kinase inhibitors, anticancer (imatinib), and others (aprepitant, casopitant, cimetidine, cyclosporine, dronedarone, grapefruit juice [citrus paradisi fruit juice], schisandra sphenanthera, tofisopam)
  - Strong CYP3A inducers: avasimibe, carbamazepine, mitotane, phenobarbital, phenytoin, rifabutin, rifampin (rifampicin), and St. John’s wort (hypericum perforatum)
- Pregnant or nursing (females of childbearing potential require a negative serum pregnancy test ≤7 days before day 1)
- Have hereditary problems of galactose intolerance, Lapp lactase deficiency, or glucose-galactose malabsorption

# Supporting Table 1. Summary of treatment-related adverse events and the most common treatment-related TEAEs by preferred term (safety population)

|  | **Dose escalation** | | | | | | | | | | **Dose expansion**^†^ | | | | | | | | | | |
| --- | --- | --- | --- | --- | --- | --- | --- | --- | --- | --- | --- | --- | --- | --- | --- | --- | --- | --- | --- | --- | --- |
|  | **Arm A: pulsed** | | | | | | **Arm B: continuous** | | | **Total** |  | | | | | | | | | | |
| n (%) | PAM 60 mg + TMZ 40 mg  7-day pulse  (n=4) | PAM 60 mg + TMZ  60 mg  7-day pulse (n=13) | PAM 60 mg  + TMZ 80 mg  7-day pulse (n=9) | PAM 60 mg + TMZ 100 mg  7-day pulse (n=3) | PAM 60 mg + TMZ  120 mg  7-day pulse (n=3) | PAM 60 mg  + TMZ 40 mg  14-day pulse (n= 14) | | PAM 60 mg + TMZ 20 mg  cont (n=14) | PAM 60 mg + TMZ 40 mg  cont (n=6) | All patients  (n=66) | Cohort 1: OC *BRCA*/HRD+ (n=4) | | | Cohort 2: TNBC *BRCA*/HRD+ (n=1) | Cohort 4: SCLC  (n=22) | | Cohort 5: gastric  (n=21) | | Cohort 6: other HRD+  (n=25) | All patients (n=73) | |
| **Patients with ≥1 treatment-related TEAE** | | | | | | | | | | | | | | | | | | | | | |
| PAM only | 2 (50.0) | 3 (23.1) | 4 (44.4) | 0 | 0 | 0 | | 1 (7.1) | 0 | 10 (15.2) | 1 (25.0) | | | 0 | 9 (40.9) | | 9 (42.9) | | 5 (20.0) | 24 (32.9) | |
| TMZ only | 2 (50.0) | 5 (38.5) | 1 (11.1) | 1 (33.3) | 1 (33.3) | 3 (21.4) | | 2 (14.3) | 1 (16.7) | 16 (24.2) | 0 | | | 1 (100) | 3 (13.6) | | 6 (28.6) | | 2 (8.0) | 12 (16.4) | |
| PAM  and TMZ | 4 (100) | 11 (84.6) | 8 (88.9) | 2 (66.7) | 3 (100) | 14 (100) | | 7 (50.0) | 5 (83.3) | 54 (81.8) | 4 (100) | | | 1 (100) | 20 (90.9) | | 18 (85.7) | | 22 (88.0) | 65 (89.0) | |
| PAM  or TMZ | 4 (100) | 12 (92.3) | 8 (88.9) | 3 (100) | 3 (100) | 14 (100) | | 8 (57.1) | 5 (83.3) | 57 (86.4) | 4 (100) | | | 1 (100) | 20 (90.9) | | 18 (85.7) | | 23 (92.0) | 66 (90.4) | |
| **Treatment-related grade 3 or higher TEAE** | | | | | | | | | | | | | | | | | | | | | |
| PAM only | 0 | 1 (7.7) | 2 (22.2) | 0 | 0 | 0 | | 0 | 0 | 3 (4.5) | 1 (25.0) | | | 0 | 6 (27.3) | | 1 (4.8) | | 1 (4.0) | 9 (12.3) | |
| TMZ only | 1 (25.0) | 0 | 0 | 0 | 0 | 1 (7.1) | | 0 | 0 | 2 (3.0) | 0 | | | 0 | 1 (4.5) | | 1 (4.8) | | 0 | 2 (2.7) | |
| PAM  and TMZ | 1 (25.0) | 6 (46.2) | 4 (44.4) | 2 (66.7) | 3 (100) | 9 (64.3) | | 6 (42.9) | 4 (66.7) | 35 (53.0) | 4 (100) | | | 1 (100) | 12 (54.5) | | 9 (42.9) | | 12 (48.0) | 38 (52.1) | |
| PAM  or TMZ | 2 (50.0) | 7 (53.8) | 5 (55.6) | 2 (66.7) | 3 (100) | 9 (64.3) | | 6 (42.9) | 4 (66.7) | 38 (57.6) | 4 (100) | | | 1 (100) | 13 (59.1) | | 10 (47.6) | | 13 (52.0) | 41 (56.2) | |
| **Treatment-related serious TEAE** | | | | | | | | | | | | | | | | | | | | | |
| PAM only | 0 | 0 | 0 | 0 | 0 | 0 | | 0 | 0 | 0 | 0 | | | 0 | 0 | | 0 | | 0 | 0 | |
| TMZ only | 1 (25.0) | 0 | 0 | 0 | 0 | 0 | | 0 | 0 | 1 (1.5) | 0 | | | 0 | 0 | | 1 (4.8) | | 0 | 1 (1.4) | |
| PAM  and TMZ | 0 | 0 | 0 | 0 | 0 | 0 | | 1 (7.1) | 0 | 1 (1.5) | 2 (50.0) | | | 0 | 4 (18.2) | | 2 (9.5) | | 2 (8.0) | 10 (13.7) | |
| PAM  or TMZ | 1 (25.0) | 0 | 0 | 0 | 0 | 0 | | 1 (7.1) | 0 | 2 (3.0) | 2 (50.0) | | | 0 | 4 (18.2) | | 3 (14.3) | | 2 (8.0) | 11 (15.1) | |
| **Treatment-related TEAE leading to death** | 0 | 0 | 0 | 0 | 0 | 0 | | 0 | 0 | 0 | 0 | | | 0 | 0 | | 0 | | 0 | 0 | |
| **Treatment-related TEAE leading to treatment discontinuation** | | | | | | | | | | | | | | | | | | | | | |
| PAM only | 0 | 0 | 0 | 0 | 1 (33.3) | 0 | | 0 | 0 | 1 (1.5) | 0 | | | 0 | 0 | | 0 | | 0 | 0 | |
| TMZ only | 0 | 0 | 0 | 0 | 0 | 0 | | 0 | 0 | 0 | 1 (25.0) | | | 0 | 0 | | 0 | | 0 | 1 (1.4) | |
| PAM  and TMZ | 0 | 0 | 0 | 1 (33.3) | 1 (33.3) | 0 | | 0 | 0 | 2 (3.0) | 1 (25.0) | | | 0 | 0 | | 1 (4.8) | | 1 (4.0) | 3 (4.1) | |
| PAM  or TMZ | 0 | 0 | 0 | 1 (33.3) | 1 (33.3) | 0 | | 0 | 0 | 2 (3.0) | 2 (50.0) | | | 0 | 0 | | 1 (4.8) | | 1 (4.0) | 4 (5.5) | |
| **Most common treatment-related to pampiparib or TMZ TEAEs by preferred term (≥20% of total)**^‡^ | | | | | | | | | | | | | | | | | | | | | |
| Anemia | 0 | 7 (53.8) | 5 (55.6) | 2 (66.7) | 3 (100) | 10 (71.4) | | 6 (42.9) | 3 (50.0) | 36 (54.5) | | 3 (75.0) | 1 (100) | | 13 (59.1) | 11 (52.4) | | 15 (60.0) | | | 43 (58.9) |
| Nausea | 3 (75.0) | 8 (61.5) | 3 (33.3) | 1 (33.3) | 1 (33.3) | 4 (28.6) | | 4 (28.6) | 3 (50.0) | 27 (40.9) | | 4 (100) | 1 (100) | | 9 (40.9) | 10 (47.6) | | 7 (28.0) | | | 31 (42.5) |
| Fatigue | 2 (50.0) | 8 (61.5) | 2 (22.2) | 0 | 0 | 9 (64.3) | | 3 (21.4) | 1 (16.7) | 25 (37.9) | | 3 (75.0) | 0 | | 8 (36.4) | 8 (38.1) | | 6 (24.0) | | | 25 (34.2) |
| Thrombocytopenia | 1 (25.0) | 5 (38.5) | 4 (44.4) | 1 (33.3) | 3 (100) | 7 (50.0) | | 1 (7.1) | 1 (16.7) | 23 (34.8) | | 2 (50.0) | 0 | | 7 (31.8) | 5 (23.8) | | 4 (16.0) | | | 18 (24.7) |
| Neutropenia | 1 (25.0) | 4 (30.8) | 3 (33.3) | 2 (66.7) | 3 (100) | 5 (35.7) | | 3 (21.4) | 1 (16.7) | 22 (33.3) | | 4 (100) | 1 (100) | | 6 (27.3) | 5 (23.8) | | 5 (20.0) | | | 21 (28.8) |
| Decreased appetite | 1 (25.0) | 4 (30.8) | 1 (11.1) | 1 (33.3) | 0 | 4 (28.6) | | 3 (21.4) | 0 | 14 (21.2) | | 3 (75.0) | 0 | | 10 (45.5) | 7 (33.3) | | 3 (12.0) | | | 23 (31.5) |
| Vomiting | 1 (25.0) | 2 (15.4) | 1 (11.1) | 0 | 1 (33.3) | 2 (14.3) | | 1 (7.1) | 1 (16.7) | 9 (13.6) | | 2 (50.0) | 0 | | 5 (22.7) | 6 (28.6) | | 2 (8.0) | | | 15 (20.5) |
| Platelet count decreased | 1 (25.0) | 0 | 3 (33.3) | 1 (33.3) | 0 | 2 (14.3) | | 3 (21.4) | 2 (33.3) | 12 (18.2) | | 1 (25.0) | 1 (100) | | 9 (40.9) | 5 (23.8) | | 8 (32.0) | | | 24 (32.9) |
| Neutrophil count decreased | 0 | 2 (15.4) | 2 (22.2) | 1 (33.3) | 0 | 2 (14.3) | | 2 (14.3) | 2 (33.3) | 11 (16.7) | | 0 | 0 | | 7 (31.8) | 4 (19.0) | | 8 (32.0) | | | 19 (26.0) |

^†^No patients were enrolled in the planned cohort 3 (metastatic castration-resistant prostate cancer). ^‡^Treatment-related TEAEs occurring in either the dose-escalation or dose-expansion stage in ≥20% of total patients.

*BRCA*, breast cancer susceptibility gene; cont, continuous; HRD+, homologous recombination deficiency; OC, ovarian cancer; PAM, pamiparib; SCLC, small cell lung cancer; TMZ, temozolomide; TNBC, triple-negative breast cancer; TEAE, treatment-emergent adverse event.

# Supporting Table 2. Dose-limiting toxicities (safety population)

|  | **Dose escalation** | | | | | | | | |
| --- | --- | --- | --- | --- | --- | --- | --- | --- | --- |
|  | **Arm A: pulsed** | | | | | | **Arm B: continuous** | | **Total** |
|  | PAM 60 mg + TMZ 40 mg  7-day pulse  (n=4) | PAM 60 mg + TMZ 60 mg  7-day pulse (n=13) | PAM 60 mg  + TMZ 80 mg  7-day pulse (n=9) | PAM 60 mg + TMZ 100 mg  7-day pulse (n=3) | PAM 60 mg + TMZ 120 mg  7-day pulse (n=3) | PAM 60 mg  + TMZ 40 mg  14-day pulse  (n= 14) | PAM 60 mg + TMZ 20 mg  Cont  (n=14) | PAM 60 mg + TMZ 40 mg  Cont  (n=6) | All patients  (n=66) |
| **Dose-limiting toxicity, n (%)** | 0 | 0 | 0 | 2 (66.7) | 2 (66.7) | 0 | 0 | 0 | 4 (6.1) |
| Neutropenia | 0 | 0 | 0 | 1 (33.3) | 2 (66.7) | 0 | 0 | 0 | 3 (4.5) |
| Neutrophil count decreased | 0 | 0 | 0 | 1 (33.3) | 0 | 0 | 0 | 0 | 1 (1.5) |

cont, continuous; PAM, pamiparib; TMZ, temozolomide.

# Supporting Table 3. Analysis of objective response rate (confirmed), GIS by *BRCA* (efficacy analysis set)

|  | Tumor BRCA mutation   (n = 7) | Tumor BRCA  wild type   (n = 35) | Tumor BRCA unknown   (n = 4) | Total   (N = 46) |
| --- | --- | --- | --- | --- |
| **GIS ≥33** |  |  |  |  |
| Objective Response Rate, % (Responders/Patients) | 50.0 (3/6) | 40.0 (2/5) | 50.0 (1/2) | 46.2 (6/13) |
| 2-sided 90% CI | 15.3 - 84.7 | 7.6 - 81.1 | 2.5 - 97.5 | 22.4 - 71.3 |
| **GIS <33** |  |  |  |  |
| Objective Response Rate, % (Responders/Patients) | 0 (0/1) | 10.0 (3/30) | 0 (0/2) | 9.1 (3/33) |
| 2-sided 90% CI | 0.0 - 95.0 | 2.8 - 23.9 | 0.0 - 77.6 | 2.5 - 21.9 |

*BRCA*, breast cancer susceptibility gene; GIS, genomic instability score.

# Supporting Table 4. Analysis of disease control rate (confirmed), GIS by *BRCA* (efficacy analysis set)

|  | Tumor BRCA mutation   (n = 7) | Tumor BRCA  wild type   (n = 35) | Tumor BRCA unknown   (n = 4) | Total   (N = 46) |
| --- | --- | --- | --- | --- |
| **GIS ≥33** |  |  |  |  |
| Disease Control Rate, % (Responders/Patients) | 100.0 (6/6) | 80.0 (4/5) | 100.0 (2/2) | 92.3 (12/13) |
| 2-sided 90% CI | 60.7 - 100.0 | 34.3 - 99.0 | 22.4 - 100.0 | 68.4 - 99.6 |
| **GIS <33** |  |  |  |  |
| Disease Control Rate, % (Responders/Patients) | 100.0 (1/1) | 53.3 (16/30) | 50.0 (1/2) | 54.5 (18/33) |
| 2-sided 90% CI | 5.0 - 100.0 | 37.0 - 69.2 | 2.5 - 97.5 | 38.9 - 69.5 |

*BRCA*, breast cancer susceptibility gene; GIS, genomic instability score.

# Supporting Table 5. Summary of pamiparib pharmacokinetic parameters^†^

|  | n/N | 60 mg BID |
| --- | --- | --- |
| AUC_0-4, SS_ (h*ng/mL) – geometric mean | 8/20 | 11,119 |
| C_max_ (ng/mL) – mean (SD) | 20/20 | 2,006 (530) |
| C_trough, SS_ (ng/mL) – mean (SD) | 14/20 | 2,934 (2346) |
| t_1/2_ (h) – mean (SD) | 17/20 | 13.5 (7.9) |
| T_max_ (h) – mean (SD) | 20/20 | 4.0 (6.7) |

^†^Parameters at steady state were determined at cycle 1, day 15.

AUC, area under the plasma concentration-time curve; BID, twice daily; C_max_, maximum observed serum concentration; C_trough_, lowest concentration reached before the next dose administered; SD, standard deviation; SS, steady state; t_1/2_, elimination half-life; T_max_, time to reach maximum (peak) plasma concentration.

# References

1. Clinical Trials Facilitation and Coordination Group. Recommendations related to contraception and pregnancy testing in clinical trials: Heads of Medicines Agencies; 2020 [updated 09/21/2020. Available from: <https://www.hma.eu/fileadmin/dateien/Human_Medicines/01-About_HMA/Working_Groups/CTFG/2020_09_HMA_CTFG_Contraception_guidance_Version_1.1_updated.pdf>

2. The Criteria Committee of the New York Heart Association. Nomenclature and Criteria for Diagnosis of Diseases of the Heart and Great Vessels. Boston: Little, Brown & Co; 1994.
